# Phytochemical Analysis of *Podospermum* and *Scorzonera n*-Hexane Extracts and the HPLC Quantitation of Triterpenes

**DOI:** 10.3390/molecules23071813

**Published:** 2018-07-21

**Authors:** Özlem Bahadır-Acıkara, Serkan Özbilgin, Gülcin Saltan-İşcan, Stefano Dall’Acqua, Veronika Rjašková, Fevzi Özgökçe, Václav Suchý, Karel Šmejkal

**Affiliations:** 1 Department of Pharmacognosy, Faculty of Pharmacy, Ankara University, Tandogan, TR-06100 Ankara, Turkey; serkan_ozbilgin@hotmail.com (S.Ö.); gulcin.saltan@pharmacy.ankara.edu.tr (G.S.-İ.); 2 Department of Pharmaceutical Sciences, University of Padua, Via Marzolo 5, I-35100 Padova, Italy; stefano.dallacqua@unipd.it; 3 Department of Natural Drugs, Faculty of Pharmacy, University of Veterinary and Pharmaceutical Sciences Brno, Palackého Třída 1946/1, CZ-61242 Brno, Czech Republic; v.rjaskova@gmail.com (V.R.); suchyv@vfu.cz (V.S.); 4 Department of Biology, Faculty of Art and Science, Yüzüncü Yıl University, TR-65080 Van, Turkey; f_ozgokce65@yahoo.com

**Keywords:** HPLC, *Podospermum*, *Scorzonera*, triterpenes

## Abstract

Previously tested *n*-hexane extracts of the *Scorzonera latifolia* showed promising bioactivity in vivo. Because triterpenes could account for this activity, *n*-hexane extracts were analyzed by HPLC to identify and quantify the triterpenes as the most abundant constituents. Other *Scorzonera* and *Podospermum* species, potentially containing triterpenic aglycones, were included in the study. An HPLC method for simultaneous determination of triterpene aglycones was therefore developed for analysis of *Podospermum* and *Scorzonera* species. *n*-Hexane extracts of root and aerial parts of *S. latifolia*, ten other *Scorzonera* species and two *Podospermum* species were studied to compare the content of triterpenes. HPLC was used for the qualitative and quantitative analysis of α-amyrin, lupeol, lupeol acetate, taraxasteryl acetate, 3-β-hydroxy-fern-7-en-6-one acetate, urs-12-en-11-one-3-acetyl, 3-β-hydroxy-fern-8-en-7-one acetate, and olean-12-en-11-one-3-acetyl. Limits of detection and quantification were determined for each compound. HPLC fingerprinting of *n*-hexane extracts of *Podospermum* and *Scorzonera* species revealed relatively large amounts of triterpenes in a majority of investigated taxa. Lupeol, lupeol acetate, and taraxasteryl acetate were found in a majority of the species, except *S. acuminata*. The presence of α-amyrin, 3β-hydroxy-fern-7-en-6-one-acetate, urs-12-en-11-one-3-acetyl, 3β-hydroxy-fern-8-en-7-one-acetate, and olean-12-en-11-one-3-acetyl was detected in varying amounts. The triterpene content could correlate with the analgesic and anti-inflammatory activity of *Scorzonera*, which was previously observed and *Scorzonera* species that have been determined to contain triterpenes in large amounts and have not yet been tested for their analgesic activity should be tested for their potential analgesic and anti-inflammatory potential. The presented HPLC method can be used for analysis of triterpene aglycones, for example dedicated to chemosystematic studies of the Scorzonerinae.

## 1. Introduction

*Scorzonera* genus belonging to Asteraceae family is widely distributed in Eurasia and northern Africa with about 160 species. In Turkey, this genus is represented by 59 taxa, and 52 species, of which 31 are endemic [1]. *Podospermum* genus (Asteraceae), represented by several tens of species, is closely related to *Scorzonera*, and also grows mainly in Mediterranean and Western Asia. Members of the *Scorzonera* genus are used as vegetables and medicinal plants. Phenolic compounds such as dihydroisocoumarins [2,3], bibenzyl derivatives [4,5,6], flavonoids [7,8,9], lignans [6,10], stilbene derivatives [11], quinic and caffeic acid derivatives [8,12], sesquiterpenes [4,8,13] and triterpenes [12,13,14,15,16,17,18] have been isolated from *Scorzonera* species. Triterpenes are one of the largest groups of terpenes [19,20]. It has been estimated that more than 4000 triterpenoids are known to occur in nature [19]. Interest in the natural triterpenoids is growing because they display a wide spectrum of biological activities [19,20,21].

The current study is aimed at developing a fingerprint profile of *n*-hexane extracts of *S. latifolia*. In addition, the triterpenes taraxasteryl acetate (**1**), 3β-hydroxy-fern-7-en-6-one-acetate (**2**), urs-12-en-11-one-3-acetyl (**3**), 3β-hydroxy-fern-8-en-7-one-acetate (**4**), and olean-12-en-11-one-3-acetyl (**5**), which have been previously isolated from the *n*-hexane extracts of *S. latifolia* and the commercially available triterpenes α-amyrin (**6**), lupeol (**7**), and lupeol acetate (**8**) have been qualitatively and quantitatively determined first in *S. latifolia*, and later in other *Scorzonera* species. Because the fingerprint profiling of the plant extracts may be useful in chemotaxonomic classification of corresponding plants and also in predicting the potential bioactivity, several aerial as well as root extracts of *Scorzonera* species collected in Turkey have been analyzed by the same method to determine their triterpene profiles and to compare their triterpene contents.

## 2. Results

**Figure 1:**
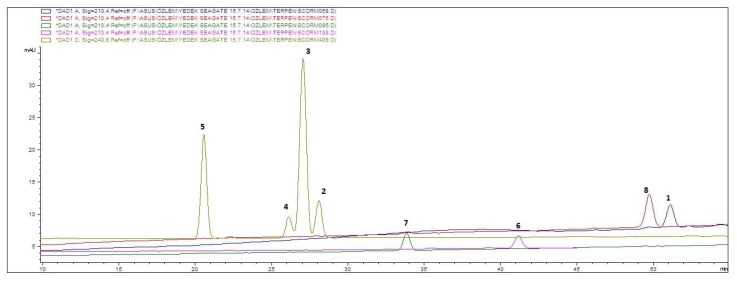
Superimposed representative HPLC chromatograms of compounds **1** and **6**–**8** at 210 nm and **2**–**5** at 240 nm: taraxasteryl acetate (**1**) 33 µg·mL^−1^; 3β-hydroxy-fern-7-en-6-one acetate (**2**) 20 µg·mL^−1^; urs-12-en-11-one-3-acetyl (**3**) 65 µg·mL^−1^; 3β-hydroxy-fern-8-en-7-one acetate (**4**) 31 µg·mL^−1^; olean-12-en-11-one-3-acetyl (**5**) 65.5 µg·mL^−1^; α-amyrin (**6**) 23 µg·mL^−1^; lupeol (**7**) 26 µg·mL^−1^; lupeol acetate (**8**) 44 µg·mL^−1^.

This paper describes the development and validation of an HPLC method for the identification of *S. latifolia* and other *Scorzonera* species in their *n*-hexane extracts as well as the quantification of the triterpenic compounds **1**–**8** in all of the *Scorzonera* and *Podospermum* species tested. The best separation of compounds **1**–**8** was obtained using a C8 stationary phase and linear gradient elution of acetonitrile in water. The absorbance at λ 200, 210 or 240 nm (Figure 1) was used to characterize the chromatogram for each compound. Table 1 shows the wavelength, calculated calibration curve, and LOD and LOQ results for each respective compound.

The results of precision tests (Table 1) indicate that the developed method is reproducible. All results demonstrated that this HPLC method is precise, reproducible and sensitive for analyzed compounds **1**–**8**.

**Figure 2:**
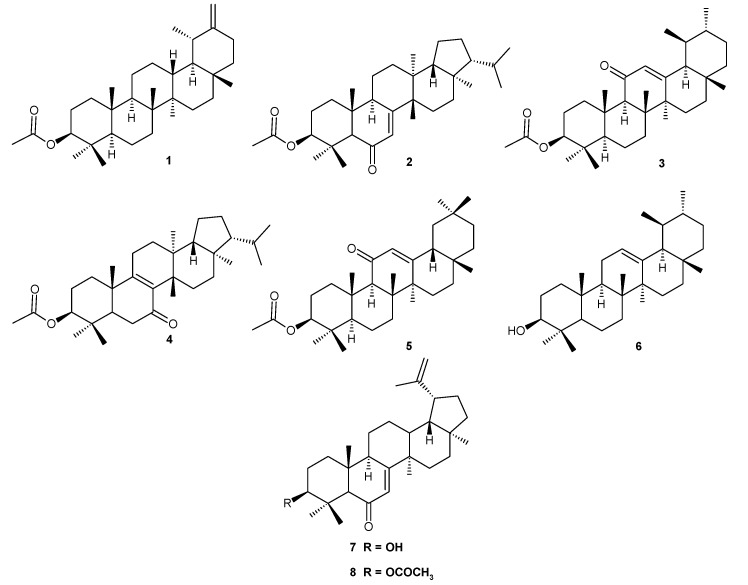
Structures of the triterpenoids **1**–**8**.

Afterwards, the roots and aerial parts of eleven different species of *Scorzonera* and two different species of *Podospermum* were subjected to extraction using *n*-hexane. The extracts were analyzed using the validated HPLC method to determine the triterpene profile and the amount of each of these triterpene aglycones (Figure 2). As shown in corresponding chromatograms presented in the Supplementary Materials, the compounds of interest were well separated in most cases (Figures S13–S38). Relatively high concentrations of taraxasteryl acetate (**1**), lupeol (**7**), and lupeol acetate (**8**) were found in the extracts of all species (with the exception of **1** in *S. acuminata*) and these compounds can therefore be referred to as major triterpenoid components of the *Scorzonera* species analyzed (Table 2). This HPLC method also enabled the qualitative and quantitative determination of **2**, which had previously been isolated from *S. latifolia* only. The minor *Scorzonera* triterpenes, 3β-hydroxy-fern-7-en-6-one acetate (**2**), urs-12-en-11-one-3-acetyl (**3**), 3β-hydroxy-fern-8-en-7-one acetate (**4**), and olean-12-en-11-one-3-acetyl (**5**), were detected, mostly in small amounts, as shown in Table 2. Although urs-12-en-11-one-3-acetyl (**3**) and 3β-hydroxy-fern-8-en-7-one acetate (**4**) were detected in the majority of the extracts, it was not possible to quantify them, even under optimal conditions.

Compounds **1**, **7**, and **8** were found in almost all of the extracts of both the aerial parts and the roots tested. The highest content of **1** was detected in the extract of the root of *S. sublanata* (4981 ± 2 µg·g^−1^), of **7** (1538± 1 µg·g^−1^) in the extract of the aerial parts of *S. latifolia*, and of **8** (4273 ± 12 µg·g^−1^) in the extract of the root of *P. canum*. Relatively high, but varying amounts of α-amyrin (**6**) were determined in *Scorzonera* species, as can be seen in Table 2. The highest content of **6** was determined to be the 3221 ± 13 µg·g^−1^ in *S. cinerea* root extract, and this, together with the high content of **8** (3645 ± 8 µg·g^−1^), **7** (1073 ± 6 µg·g^−1^), and **1** (2171 ± 6 µg·g^−1^), showed that the root of this *Scorzonera* species has the richest content of the triterpene aglycones monitored. The lowest triterpenoid content was determined in *S. sublanata* aerial parts with 338 ± 6 µg·g^−1^, 169 ± 1 µg·g^−1^, and 302 ± 1 µg·g^−1^ for compound taraxasteryl acetate (**1**), lupeol (**7**) and lupeol acetate (**8**), respectively. Total triterpenoid contents of the roots of investigated species were found to be higher than aerial parts. *S. acuminata* roots and aerial parts did not contain taraxasteryl acetate (**1**) and lupeol acetate (**8**) was detected in low amount (297 ± 1 µg·g^−1^ and 67 ± 1 µg·g^−1^). On the other hand, content of α-amyrin (**6**) was determined in relatively high amount as 1646 ± 10 µg·g^−1^ and 1102 ± 6 µg·g^−1^ for roots and aerial parts of *S. acuminata*, respectively.

## 3. Discussion

To our best knowledge, this is the first report of triterpenes in *P. canum*, *P. laciniatum*, *S. acuminata*, *S. eriophora*, *S. incisa*, *S. mirabilis*, *S. mollis*, *S. parviflora*, *S. suberosa*, and *S. sublanata*. Different triterpenes were previously isolated from other *Scorzonera* species: oleanane and ursane type from *S. austriaca* [18], and *S. mongolica* [17]; dammarane and tirucallane triterpenes from *S. divaricata* [13]; and β-amyrin, methyl oleate and methyl ursolate from *S. undulata* subsp. *deliciosa* [16]. Jehle et al. [12] described 3α-hydroxyolean-5-ene, lupeol (**7**), and magnificol in *S. aristata*. *S. mongolica* is a source of erythrodiol and moradiol (oleane derivatives) [17]. We here revealed that all tested *Podospermum* and *Scorzonera* species contain taraxasteryl acetate (**1**) except of *S. acuminata*. All *Podospermum* and *Scorzonera* species investigated here were also found to contain **7** and **8** in varying amounts. α-Amyrin (**1**), olean-12-en-11-one-3-acetyl (**5**), urs-12-en-11-one-3-acetyl (**3**), and two fernane derivatives 3β-hydroxy-fern-7-en-6-one-acetate (**2**), and 3β-hydroxy-fern-8-en-7-one-acetate (**4**) were detected here in varying amounts depending on the *Podospermum* and *Scorzonera* species as minor components. The method used allowed us to simply identify and quantify main triterpenic compounds in all *Podospermum* and *Scorzonera* species tested, and accordingly would be useful for work on other species.

Triterpenes are compounds distributed broadly in plant kingdom, with approximately 200 different skeletons showing great variability and diversity of triterpene metabolism [22]. Hill and Connoly reviewed the progress in triterpene isolation from 2012 [23]. More than 700 different triterpenes were described in this review, showing number of plant species as sources of various triterpenes and enormous progress in area of triterpene phytochemistry. Some triterpenoids can possess chemotaxonomic importance, as shown for example for triterpenes isolated from Conifers [24]. Interestingly, comparison of triterpenes which are present in soil and sediments with literature survey of triterpenes in Asteraceae showed the possibility of chemosystematic usage of some acetylated triterpenes [25]. Our analysis identified five types of triterpenes: taraxastane (**1**), ursanes (**3** and **6**), oleananes (**5**), lupanes (**7** and **8**), and fernanes (**2** and **4**), with six of the eight isolated compounds acetylated at the position 3 of the skeleton. However, the number of profiled species and identified compounds is still low.

Some attempts to give an overview of phytochemicals and chemosystematic analysis of Asteraceae were performed in the past, with a focus on presence of sesquiterpenic lactones, pyrrolizidine alkaloids, and polyacetylenes, and on the occurrence of in general highly oxidized compounds, which could be a structural feature shared by the majority of the Asteraceae chemicals [26,27]. Further studies showed phenolics as possible chemosystematic markers for the Asteraceae family (Cichoriae tribe) and for the Scorzonerinae subtribe in particular [28,29]. The work of Calabria [26] also tried to evaluate the presence of triterpenes in Asteraceae, showing their presence in 28 of 35 tribes of this family that time analyzed, 19 occurrences in Cichorieae. However, the information about the presence of triterpenes, and even the specific compounds, is still limited. Therefore, a reliable method for routine extraction and chromatographic analysis of triterpenic profile would be valuable not only for taxonomic evaluation of *Podospermum* and *Scozonera*, but also for other Cichorieae taxa and Asteraceae in general.

In Turkey and in some European countries, *Scorzonera* species are used mainly as a vegetable food [30,31]. However, the ethno-medicinal importance of the genus in Turkish, European, Chinese, Mongolian, and Libyan folk medicines has been reported [6,11,32,33,34]. Turkish folk medicine uses *Scorzonera* preparations to treat a variety of illnesses, including inflammation [35]. Triterpenic compounds are, besides inflammation, often connected with cytotoxicity and anticancer potential, as reviewed, for example, for lupeol derivatives [36,37,38,39,40,41]. For other triterpenes, cytotoxic triterpenes have previously been isolated from *S. divaricata* and *S. hispanica*. Furthermore, analgesic, anti-inflammatory, and wound healing activities of *Scorzonera* species have been reported by in vivo tests [15,42,43,44,45,46]. Some compounds responsible for the analgesic activity have been isolated by bioassay-guided fractionation from *n*-hexane extract of *S. latifolia* and identified as taraxasteryl acetate (**1**), taraxasteryl myristate [15], motiol and β-sitosterol [43]. *n*-hexane extract displayed higher activity than these isolated compounds, therefore analgesic activity of the *Scorzonera* extracts is suggested from possible synergistic interaction of the other triterpenes [15,45,46,47,48]. The same could be valid for anti-inflammatory activity of *Scorzonera*, and taraxasteryl acetate (**1**) [47,48], α-amyrin (**6**) [49,50], lupeol (**7**) [51], and lupeol acetate (**8**) [52], as visible from previously published studies anti-inflammatory active, could contribute to the antiphlogistic effect of *Scorzonera* species. Lupeol (**7**), which is relatively commonly found in several plant species, is reported to exhibit many kinds of biological activities, including anticancer, antiprotozoal, chemopreventive, and anti-inflammatory activities [53]. The anti-inflammatory activity of **7** is reportedly accompanied by immune modulatory and antitumor properties [39,41,51]. Compound **8** has demonstrated anti-inflammatory activity by regulating TNF-α and IL-2 specific mRNA and up-regulating the synthesis of IL-10 mRNA [40]. Taraxasteryl acetate (**1**), which is identified in all *Scorzonera* species investigated in concentrations ranging between 66.52 ± 1.0 and 4272.63 ± 11.61 µg·g^−1^, has been reported to have anti-inflammatory and analgesic activities [43,48]. Analgesic, anti-inflammatory, and wound healing activities of *Scorzonera* species have been reported by in vivo tests [15,42,44,45,46,47]. Some compounds responsible for the analgesic activity have been isolated by bioassay-guided fractionation from *n*-hexane extract of *S. latifolia* and identified as taraxasteryl acetate (**1**), taraxasteryl myristate [15], motiol and β-sitosterol [44] *n*-hexane extract displayed higher activity than these isolated compounds, therefore analgesic activity of the *Scorzonera* extracts is suggested from possible synergistic interaction of the other triterpenes [15,44]. According to the current study results, triterpene content of the *S. tomentosa* is found to be higher than *S. latifolia* which is followed by *S. mollis* subsp. *mollis* and *S. suberosa* ssp. *suberosa* roots. Analgesic activities of these mentioned species seem to be correlated with their triterpene contents, as we reported in our previous studies. *S. tomentosa, S. latifolia, S. mollis* subsp. *mollis* and *S. suberosa* ssp. *suberosa* roots displayed antinociceptive activities in acetic acid induced writhing test [47]. Therefore, these results encourage us to conduct further investigation, testing analgesic and anti-inflammatory activities of the species characterized by higher triterpene content, such as *S. cinerea*, *S. sublanata*, and *S. cana* var. *jacquiniana*.

## 4. Materials and Methods

### 4.1. Plant. Material

*Podospermum* and *Scorzonera* species were collected in different parts of Turkey. The plants were collected during flowering period, in eleven specimens. The taxonomic identification of the plants was confirmed by Prof. Hayri Duman, a plant taxonomist at the Department of Biological Sciences, Faculty of Sciences, Gazi University, Ankara, Turkey. Flora of Turkey and The East Aegean Islands was used for identification [54]. Voucher specimens were placed in the herbarium at the Faculty of Pharmacy of Ankara University (Table 3). The pictures of collected plant materials are available in the Supplementary Materials (Figures S1–S12). Basic rules for consistent characterization and documentation of plant source materials were followed [55].

### 4.2. HPLC Analysis

#### 4.2.1. Optimization of Sample Extraction Procedure and Preparation of Samples

Air-dried and powdered aerial parts and roots (1 g for each) (homogenized mixtures of ten specimens) of the selected *Podospermum* and *Scorzonera* species (Table 3) were used for the extraction procedures. *n*-Hexane (50 mL per 1 g of plant material), petroleum ether, chloroform, and diethylether were tested for extraction of plant material. All prepared extracts were analyzed by HPLC and *n*-hexane was found to be a more suitable solvent than other solvents for extracting the triterpene aglycones found in *Scorzonera* species because the areas of peaks were greater than petroleum ether, and the selectivity was better than that of chloroform and diethylether, which allowed us to extract some flavonoids and isocoumarins complicating the chromatogram evaluation. Furthermore, triterpenes in chloroform and diethylether extracts were observed only as minor components. After selection of solvent, temperature was used as variable factor affecting extraction. Tests at 24 °C (room temperature), 50 °C, and 69 °C (the boiling temperature of *n*-hexane) were used to determine the most suitable conditions for the extraction procedure. *n*-Hexane at room temperature (24 °C) allowed us to extract the triterpenes more selectively than at higher temperatures. The extraction time was set to 8 h, using continuous stirring. Finally, 50 mL of *n*-hexane were used to prepare extract (1 g of plant material). Each prepared extract was later evaporated to dryness and the residual solids were dissolved in isopropanol (Merck, Darmstadt, Germany) and adjusted into 10 mL volumetric flasks. Each solution was filtered through a 0.45 μm membrane filter before injection.

#### 4.2.2. Optimization of Conditions for HPLC Analysis

An HPLC method was developed to analyze the triterpenoids in *Scorzonera* extracts. An Agilent model LC 1100 chromatograph (Agilent Technologies, Santa Clara, CA, USA) equipped with a DAD (diode array detector) was used. The DAD was set to a wavelength of 200 or 240 nm. The chromatograms were analyzed and the peak areas integrated automatically using Agilent ChemStation Software.

Waters Spherisorb S5W normal-phase (25 cm × 4 mm, 5 µm), Supelcosil C18 reversed-phase (25 cm × 4 mm, 5 µm), and ACE 5 C8 reversed-phase (25 cm × 4.6 mm, 5 μm) columns were tested to obtain optimal separation. Methanol (HiPerSolv Chromanorm 20,864.320, VWR, Leuven, Belgium), acetonitrile (Merck 1.00030.2500, Darmstadt, Germany), and water (Extra pure water obtained from Millipore Milli Q Gradient A10, Milford, MA, USA) were used in different proportions as components of the mobile phase. Series of experiments showed the optimal separation to be achieved by using the ACE 5 C8 column with acetonitrile water gradient elution, with water (A) and acetonitrile (B) in a linear gradient elution: the initial composition at time 0 A:B 20:80 (*v*/*v*), after 70 min of following a linear gradient, was changed to A:B 6:94 (*v*/*v*), and after 70 min 100% B to wash column. The flow rate was 0.8 mL·min^−1^. The column temperature was maintained at 40 °C, and the sample injection volume was 10 μL.

#### 4.2.3. Preparation and Calibration of Standard Solutions

Stock solutions of compounds **1**–**5** obtained from *Scorzonera* species [15,56] and **6**–**8** (from Sigma-Aldrich, St. Louis, MI, USA) were weighed and dissolved in isopropanol (Merck, Darmstadt, Germany). The purity of isolated compounds was determined from HPLC analysis (>98%) and ^1^H NMR analysis. Six different concentrations of each compound were prepared in the following ranges: 16.5-330 µg·mL^−1^ for taraxasteryl acetate (**1**), 8–160 µg·mL^−1^ for 3β-hydroxy-fern-7-en-6-one acetate (**2**), 26–520 µg·mL^−1^ for urs-12-en-11-one-3-acetyl (**3**), 12.5–250 µg·mL^−1^ for 3β-hydroxy-fern-8-en-7-one acetate (**4**), 25–500 µg·mL^−1^ for olean-12-en-11-one-3-acetyl (**5**), 11.5–230 µg·mL^−1^ for α-amyrin (**6**), 13–260 µg·mL^−1^ for lupeol (**7**), and 22–440 µg·mL^−1^ for lupeol acetate (**8**). Injections of 10 µL were performed in triplicate for each concentration of each standard solution. The area of the peak resulting from each injection was plotted against the known concentration of the substance to obtain the calibration curve.

#### 4.2.4. Validation Procedure

##### Limits of Detection and Quantification

Standard HPLC validation procedures [57] were used to determine the limits of detection and quantification (LOD and LOQ), respectively. The LOD and LOQ were established at signal to noise ratios (S/N) of 3 and 10, respectively (Table 1). The LOD and LOQ concentrations were verified experimentally by repeating each analysis six times.

##### Precision

Intra-day precision tests were performed by analyzing the same standard solutions of all compounds at the LOQ level six times in a single day. Inter-day precision tests were performed by analyzing standard solutions at three different concentrations on three different days, respectively. The results of precision tests (Table 1) indicate that the developed method is reproducible. All results demonstrated that this HPLC method is precise, reproducible and sensitive.

## 5. Conclusions

An HPLC method for the identification and quantification of the triterpenes found in species of the genus *Scorzonera* was developed in the current study. This method allows establishing fingerprint chromatograms of *n*-hexane extracts of *Scorzonera* and *Podospermum* and quantifying taraxasteryl acetate (**1**), α-amyrin (**6**), lupeol (**7**), and lupeol acetate (**8**) as major triterpenes, and 3β-hydroxy-fern-7-en-6-one acetate (**2**), urs-12-en-11-one-3-acetyl (**3**), 3β-hydroxy-fern-8-en-7-one acetate (**4**), and olean-12-en-11-one-3-acetyl (**5**) as minor triterpenes. The amounts of the triterpenes, especially α-amyrin (**6**), lupeol (**7**), lupeol acetate (**8**), and taraxasteryl acetate (**1**), found in the different species could correlate with the analgesic and anti-inflammatory activity previously observed for preparations made from these plants. Further studies confirming this correlation are necessary.

## Figures and Tables

**Table 1:** Calibration curves, linearity, LOD, LOQ and precision of HPLC analysis for triterpenes **1**–**8**.

Compound	Calibration Curve	*r* ^2^	LOD (µg/mL)	LOQ (µg/mL)	Precision %	
					Intra-Day (*n* = 6)	Inter-Day (*n* = 3)
**1**	Y = 5.1753X − 1.86223	0.9977	4.69	15.63	0.098	1.690
**2**	Y = 8.5973X + 24.3984	0.9999	1.03	3.43	0.021	0.144
**3**	Y = 3.1778X + 2.6354	0.9998	2.13	7.10	0.140	0.276
**4**	Y = 12.8099X + 61.2355	0.9969	1.04	3.47	0.037	0.176
**5**	Y = 7.2502X − 33.5294	0.9942	1.80	6.00	0.039	0.099
**6**	Y = 6.0380X + 6.2415	0.9996	0.84	2.68	0.176	0.088
**7**	Y = 6.1333X − 10.0885	0.9988	1.69	5.63	0.082	2.747
**8**	Y = 7.3958X − 24.749	0.9993	2.84	9.46	0.060	2.525

X: Concentration of compound **1**–**8** (μg/mL), Y: area under the curve.

**Table 2:** Quantification of triterpenoids **1**–**8**.

Species	Root or Aerial Part	Compound Content (µg·g^−1^; Calculated for Dry Weight of Plant Material)								
		1	2	3	4	5	6	7	8	Total Content
***P. canum*** (syn. *S. cana* var. *jacquiniana*)	R	719 ± 3	n.d.	tr.	n.d.	tr.	920 ± 11	932 ± 2	4273 ± 12	6844
	AE	81 ± 3	n.d.	tr.	n.d.	tr.	442 ± 5	932 ± 2	535 ± 4	1991
***P. laciniatum*** (syn. *S. laciniata* subsp. *laciniata*)	R	276 ± 3	n.d.	tr.	n.d.	tr.	146 ± 4	447 ± 2	3212 ± 13	4081
	AE	69 ± 5	n.d.	tr.	n.d.	tr.	209 ± 3	1025 ± 6	892 ± 2	2195
***S. acuminata***	R	n.d.	n.d.	n.d.	n.d.	n.d.	1646 ± 10	512 ± 1	297 ± 1	2456
	AE	n.d.	n.d.	n.d.	n.d.	n.d.	1102 ± 6	327 ± 5	67 ± 1	1496
***S. cinerea***	R	2171 ± 6	65 ± 1	tr.	tr.	115 ± 1	3221 ± 13	1073 ± 6	3645 ± 8	10,290
	AE	417 ± 11	tr.	tr.	tr.	tr.	309 ± 2	1174 ± 16	839 ± 6	2738
***S. eriophora***	R	3212 ± 17	20 ± 1	tr.	tr.	tr.	n.d.	244 ± 7	2195 ± 7	5672
	AE	545 ± 5	tr.	tr.	tr.	tr.	n.d.	228 ± 6	368 ± 1	1142
***S. incisa***	R	1191 ± 5	n.d.	tr.	tr.	151 ± 1	n.d.	283 ± 2	736 ± 10	2362
	AE	280 ± 10	n.d.	tr.	n.d.	n.d.	644 ± 2	1090 ± 2	236 ± 9	2250
***S. latifolia***	R	4201 ± 16	50 ± 1	tr.	tr.	135 ± 1	n.d.	213 ± 2	2261 ± 94	6861
	AE	1062 ± 2	18 ± 1	tr.	tr.	tr.	827 ± 2	1538 ± 1	607 ± 1	4051
***S. mirabilis***	R	2099 ± 4	tr.	n.d.	tr.	n.d.	n.d.	224 ± 1	1356 ± 2	3678
	AE	1262 ± 728	tr.	tr.	tr.	tr.	n.d.	954 ± 14	998 ± 13	3214
***S. mollis*** subsp. ***szowitsii***	R	3791 ± 14	n.d.	tr.	tr.	tr.	609 ± 6	282 ± 11	1244 ± 1	5926
	AE	263 ± 4	n.d.	tr.	n.d.	n.d.	246 ± 8	321 ± 1	149 ± 7	979
***S. parviflora***	R	811.96 ± 4	n.d.	tr.	tr.	tr.	n.d.	132 ± 4	711 ± 3	1656
	AE	433 ± 2	n.d.	tr.	tr.	tr.	n.d.	649 ± 6	594 ± 5	1676
***S. suberosa*** subsp. ***suberosa***	R	2340 ± 6	n.d.	tr.	tr.	tr.	n.d.	342 ± 4	1261 ± 5	3943
	AE	535 ± 4	n.d.	tr.	n.d.	tr.	n.d.	1005 ± 17	312 ± 4	1853
***S. sublanata***	R	4981 ± 2	35 ± 1	tr.	tr.	tr.	n.d.	415 ± 1	3920 ± 8	9351
	AE	338 ± 6	tr.	tr.	tr.	n.d.	n.d.	169 ± 1	302 ± 1	809
***S. tomentosa***	R	3168 ± 12	47 ± 1	tr.	tr.	187 ± 1	969 ± 11	564 ± 2	2502 ± 7	7435
	AE	376 ± 13	tr.	tr.	tr.	tr.	tr.	509 ± 2	411 ± 1	1296

Taraxasteryl acetate (**1**), α-amyrin (**6**), lupeol (**7**), lupeol acetate (**8**) (at 200 nm); and of 3β-hydroxy-fern-7-en-6-one-acetate (**2**), urs-12-en-11-one-3-acetyl (**3**), 3β-hydroxy-fern-8-en-7-one-acetate (**4**), and olean-12-en-11-one-3-acetyl (**5**) (at 240 nm) measured in μg·g^−1^. R, root; AE, aerial part; tr., traces (<LOQ level); n.d., not detected. The value was calculated as average of three independent measurements, with SD. Total content of triterpenes was calculated as a sum of amounts for compound **1**–**8.**

**Table 3:** Location of plant sample collection and the corresponding voucher specimen number.

Plant Species of the Genus *Scorzonera*	Collection Locality and Coordinates	Herbarium No.
*P. canum* C. A. Meyer, (syn. *S. cana* (C.A. Meyer) Hoffm. var. *jacquiniana* (W. Koch) Chamberlain)	Ankara, Çamlıdere N 40°29′15.695″ E 32°28′9.862″	AEF 23834
*P. laciniatum* (L.) DC. (syn. *S. laciniata* L. subsp. *laciniata*)	Ankara, Çamlıdere N 40°29′15.695″ E 32°28′9.862″	AEF 23835
*S. acuminata* Boiss.	Çankırı, Yumaklı Village N 40°26′7.103″ E 32°45′41.665″	AEF 25938
*S. cinerea* Boiss.	Sivas, Çetinkaya N 39°15′26.566″ E 37°38′7.844″	AEF 23829
*S. eriophora* DC.	Ankara, Çubuk N 40°14′13.492″ E 33°01′52.513″	AEF 23832
*S. incisa* DC.	Konya, Ermenek N 36°38′3.351″ E 32°53′32.567″	AEF 23833
*S. latifolia* (Fisch. & Mey.) DC.	Kars, Arpaçay N 40°54′57.305″ E 43°21′2.969″	AEF 23830
*S. mirabilis* Lipschitz	Van N 38°29′59.3412″ E 43°22′41.3148″	F 18386
*S. mollis* Bieb. subsp. *szowitsii* (DC.) Chamberlain	Ankara, Kızılcahamam N 40°26′49.009″ E 32°37′6.269″	AEF 23844
*S. parviflora* Jacq.	Ankara, Gölbaşı N 39°48′19.8″ E 32°48′10.799″	AEF 25894
*S. suberosa* C. Koch subsp. *suberosa*	Kayseri, Pınarbaşı N 38°42′55.868″ E 36°24′26.345″	AEF 23843
*S. sublanata* Lipschitz	Ankara, Kızılcahamam N 39°39′43.223″ E 35°51′40.547″	AEF 25937
*S. tomentosa* L.	Yozgat, Akdağmadeni N 40°28′13.253″ E 32°39′0.73″	AEF 23841

## Supplementary Materials

The following are available online, Figures S1–S12: Pictures of voucher specimens, Figures S13–S38: Chromatograms of analyzed extracts.
