# Supplementary material for: Phytochemical Analysis of Podospermum and Scorzonera n-Hexane Extracts and the HPLC Quantitation of Triterpenes
Source: Molecules. 2018 Jul 21;23(7):1813. doi: 10.3390/molecules23071813 (PMC6100500; doi:10.3390/molecules23071813)
Supplement: Supplementary file 1 [file molecules-23-01813-s001.pdf]

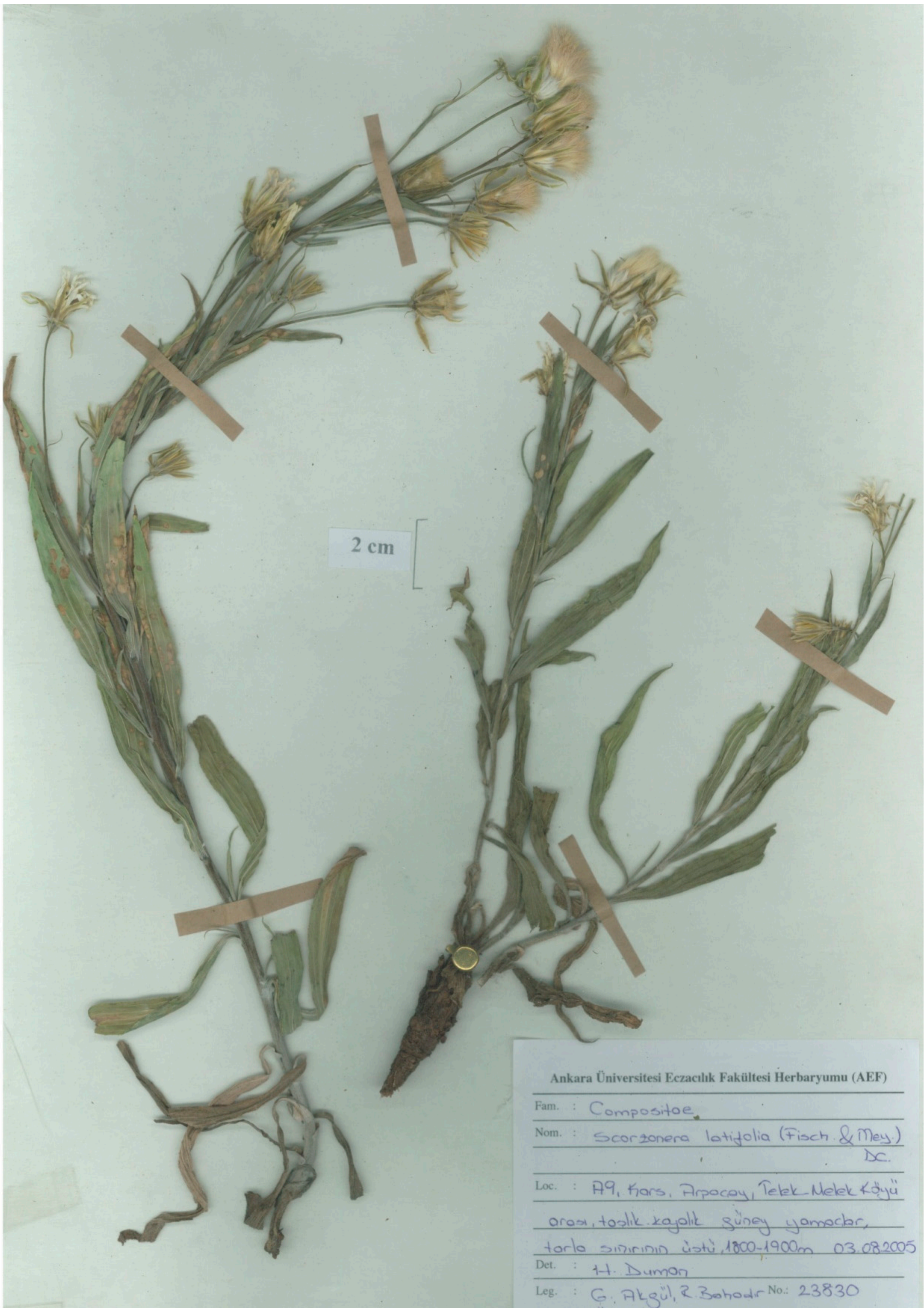

Figure S1: *Scorzonera latifolia* (Fisch&Mey) DC.

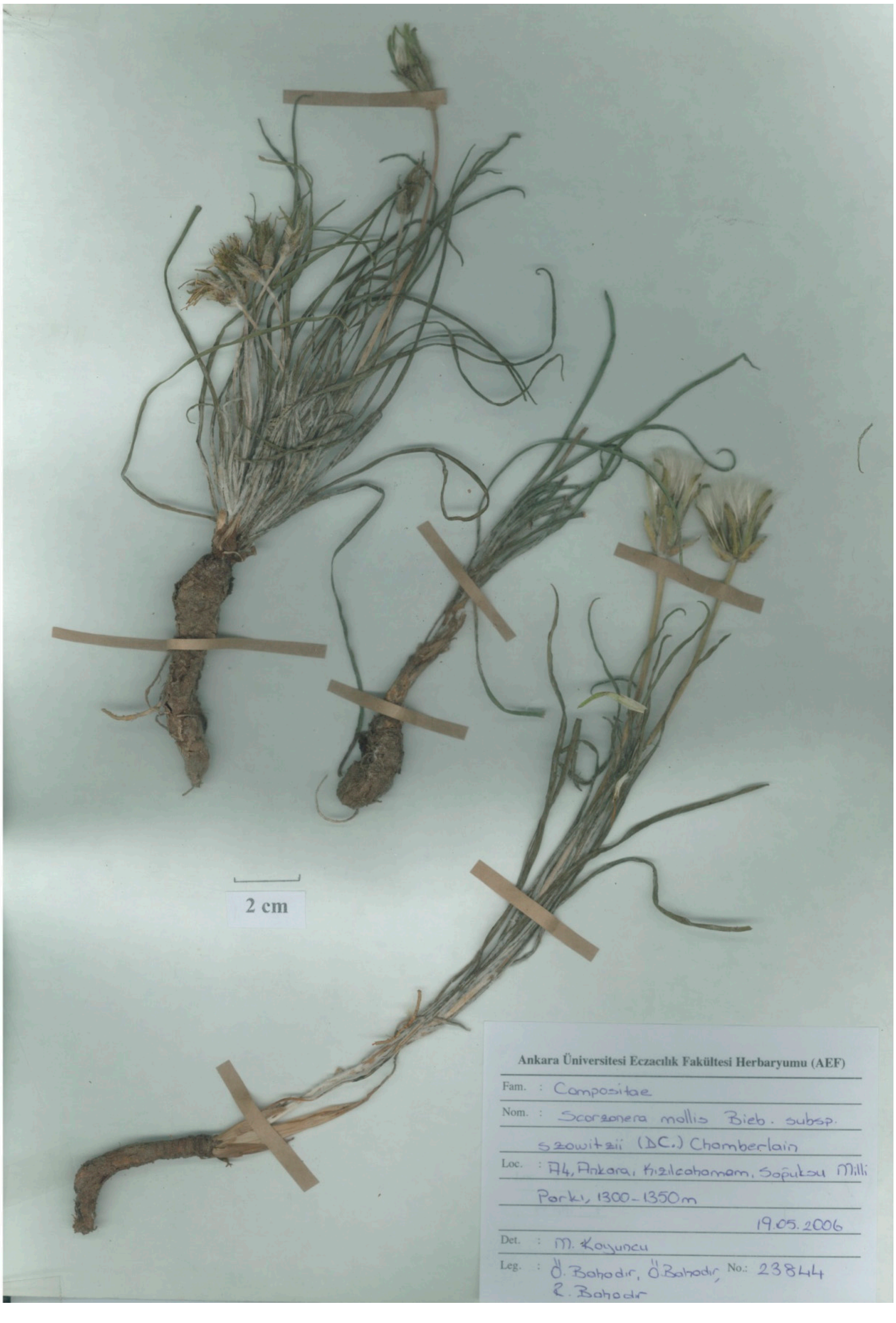

Figure S2: *Scorzonera mollis* Bieb. ssp. *szowitsii*

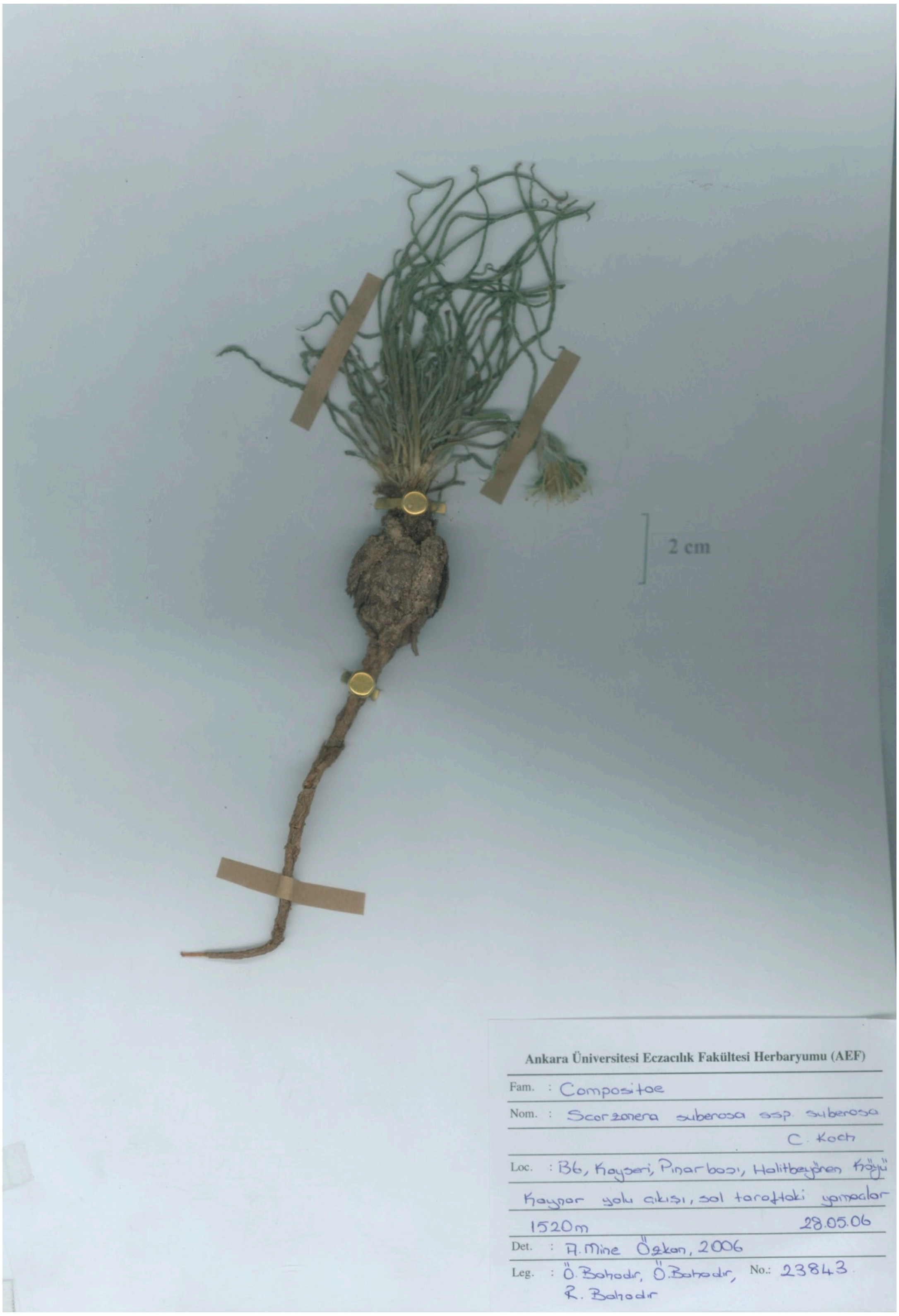

Figure S3: *Scorzonera suberosa* ssp. *suberosa* C. Koch

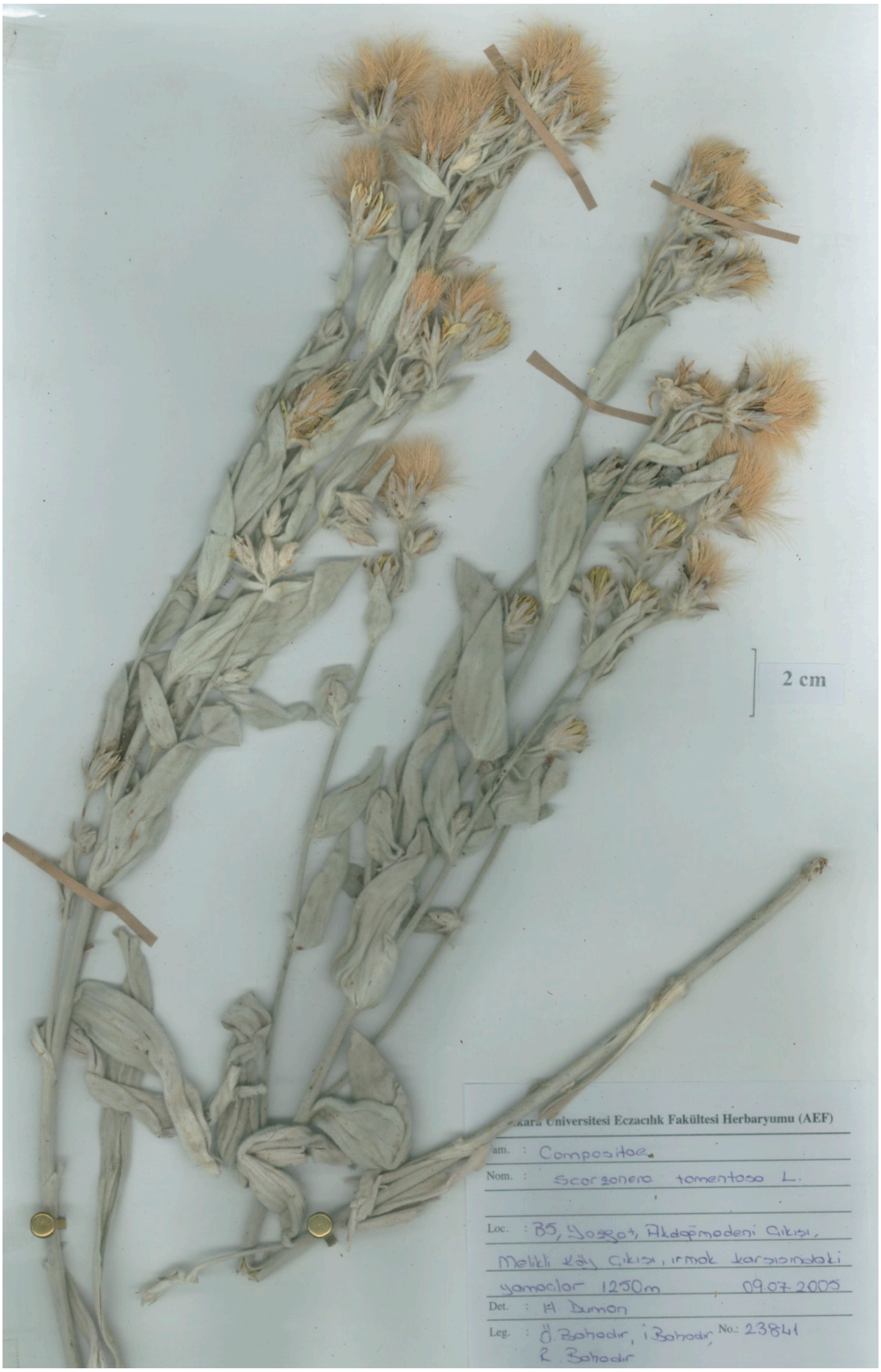

Figure S4: *Scorzonera tomentosa* L.

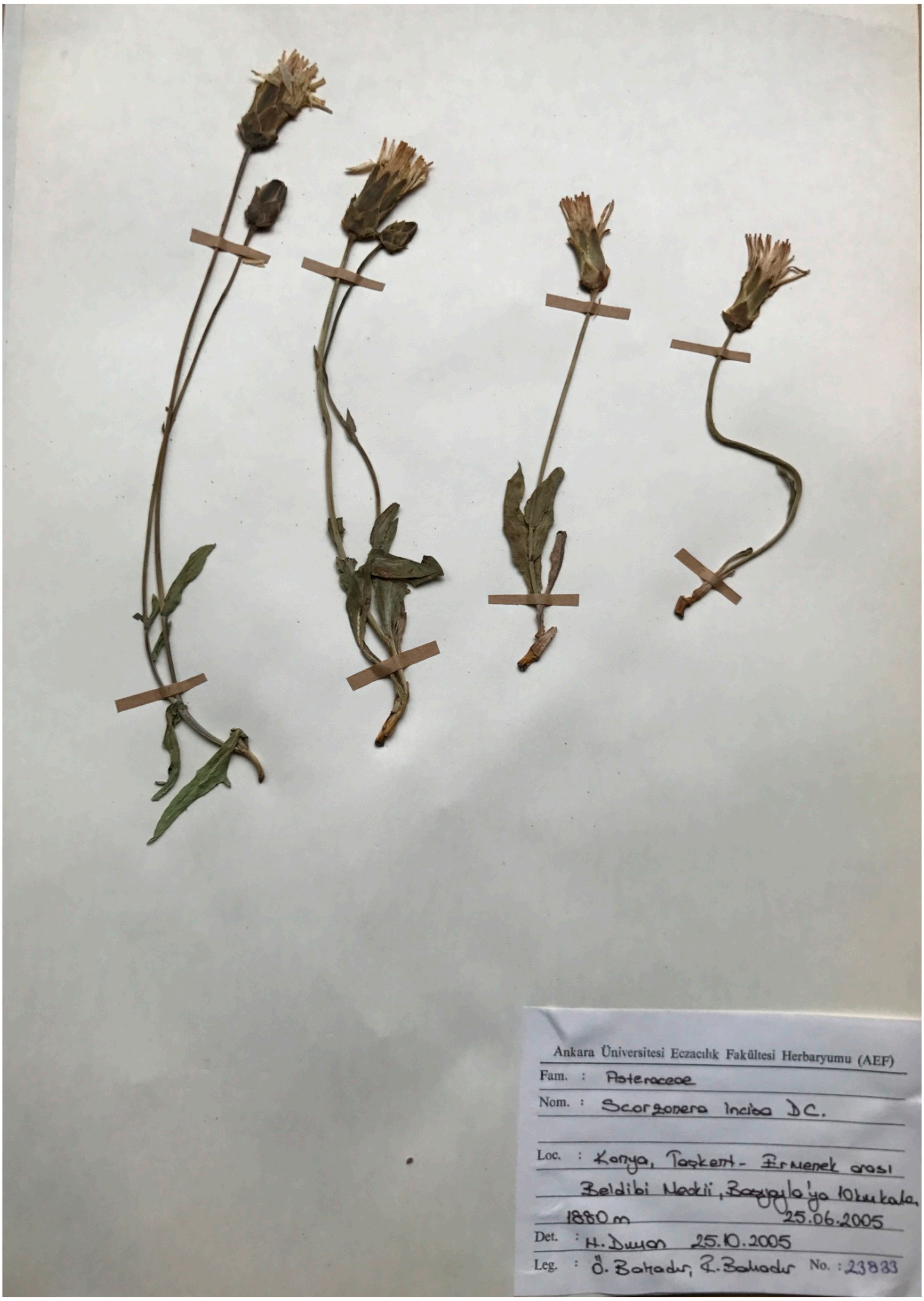

Figure S5: ***Scorzonera incisa*** **DC.**

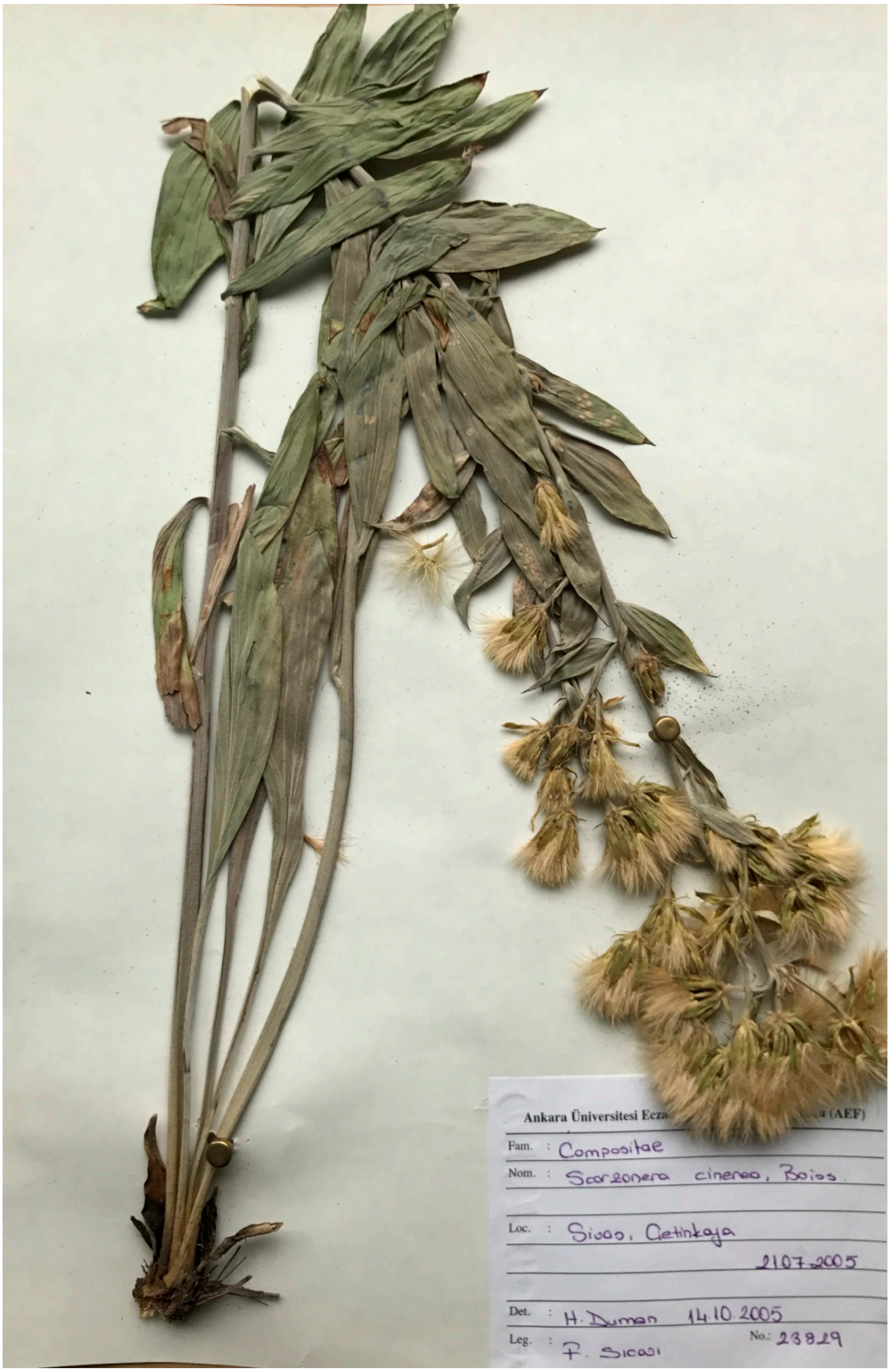

Figure S6: ***Scorzonera cinerea*** **Boiss.**

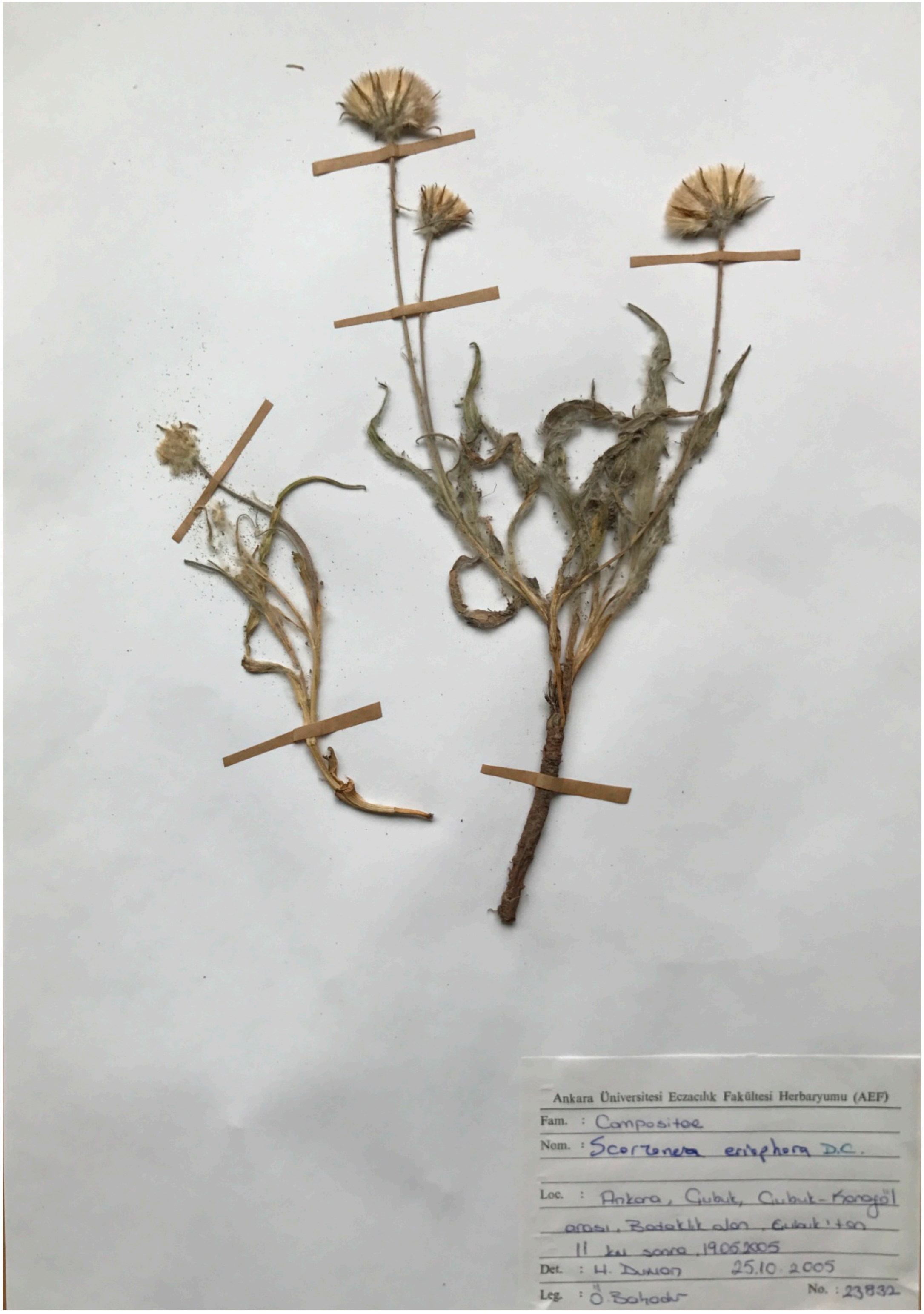

Figure S7: ***S. eriophora*** **DC.**

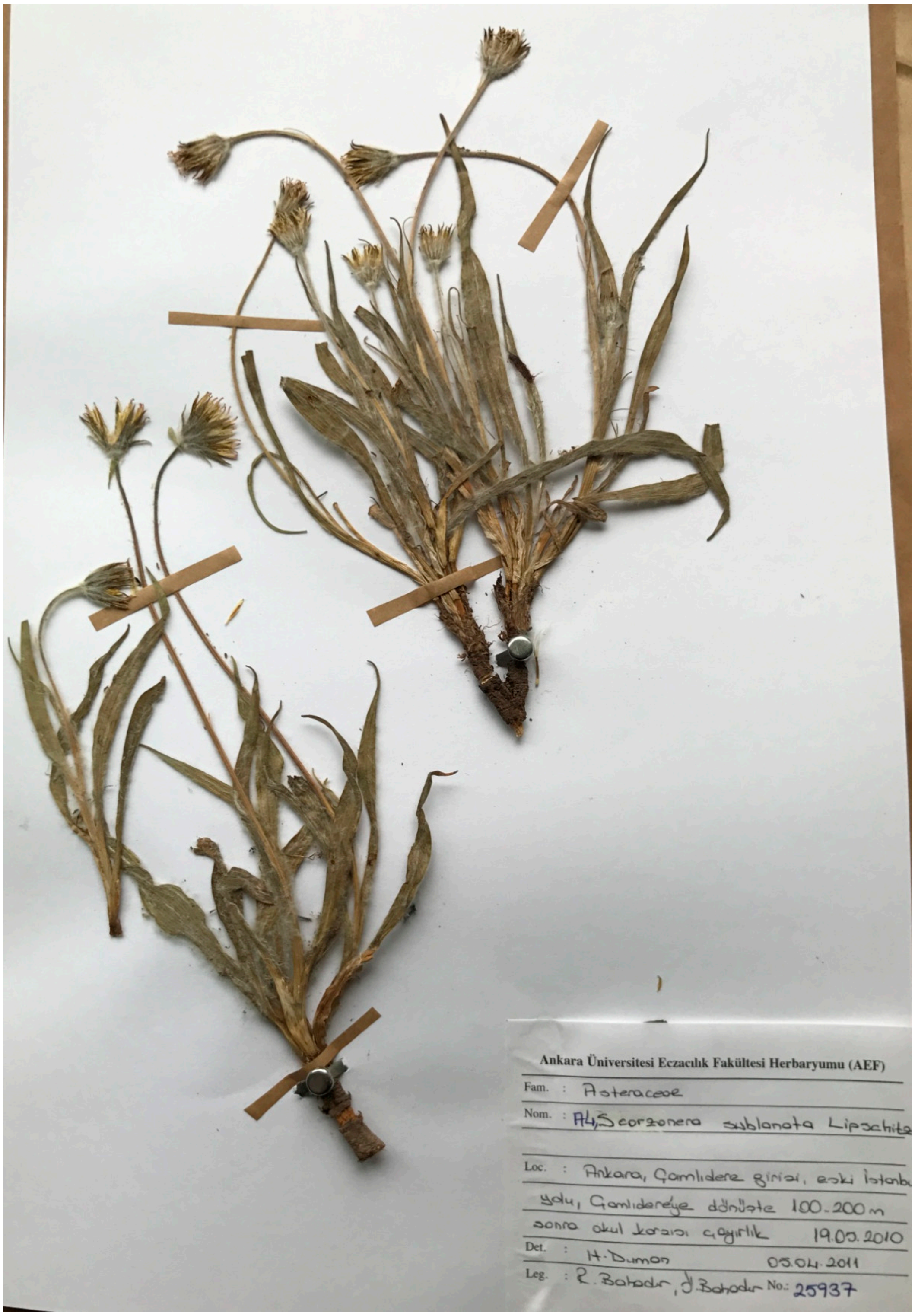

Figure S8: *Scorzonera sublanata* Lipschitz

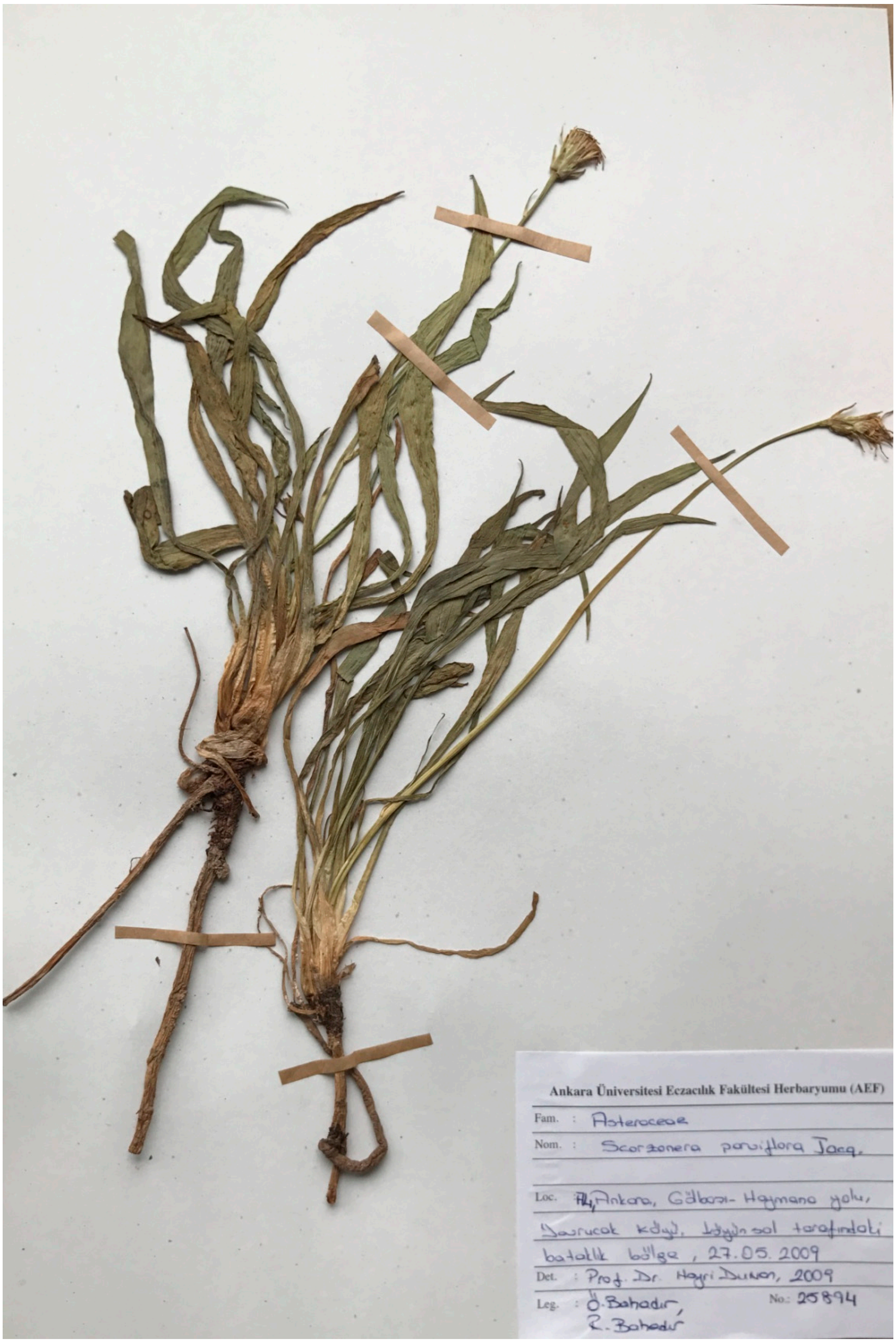

Figure S9: ***Scorzonera parviflora*** **Jacq.**

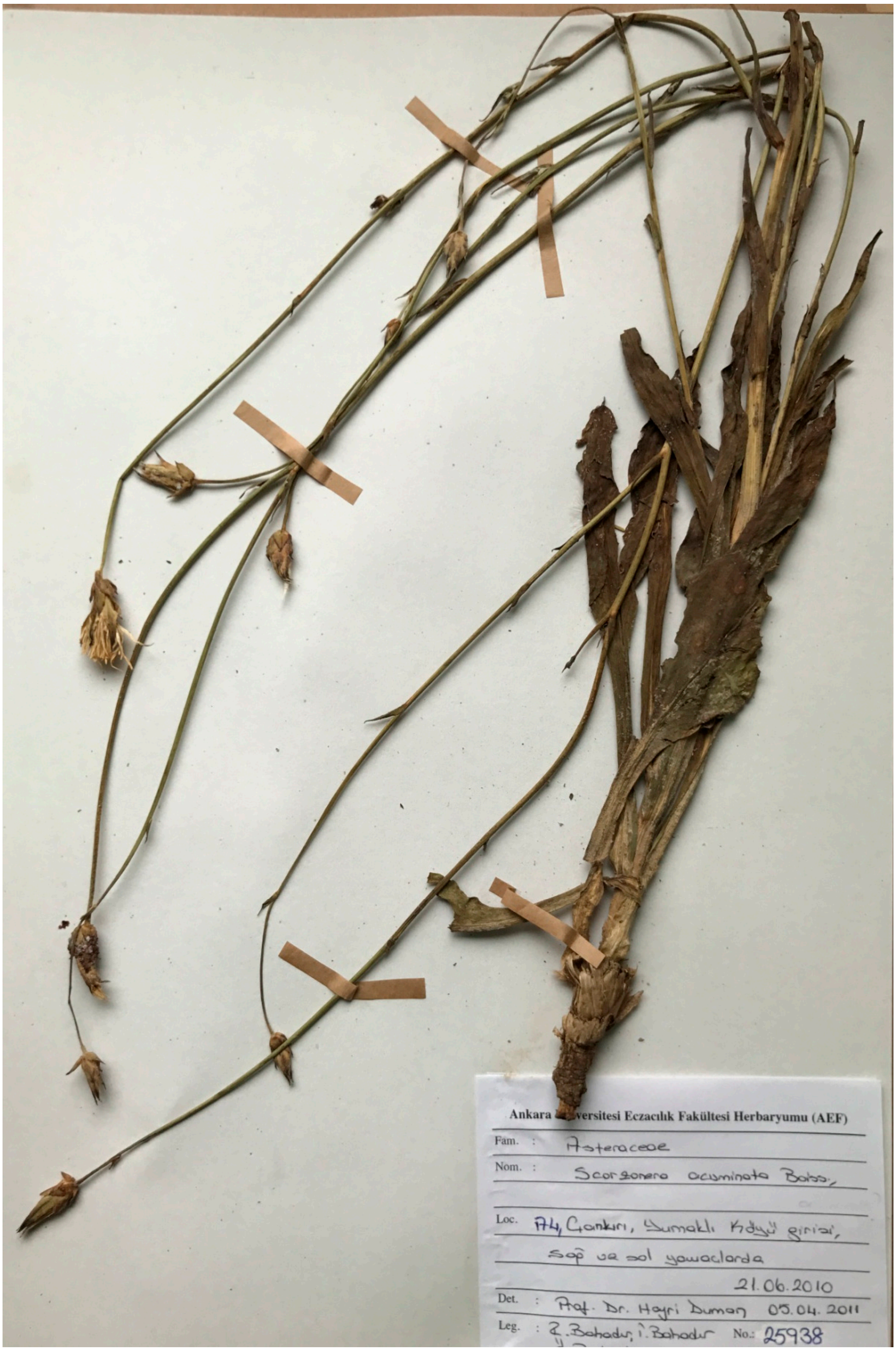

Figure S10: ***Scorzonera acuminata*** **Boiss.**

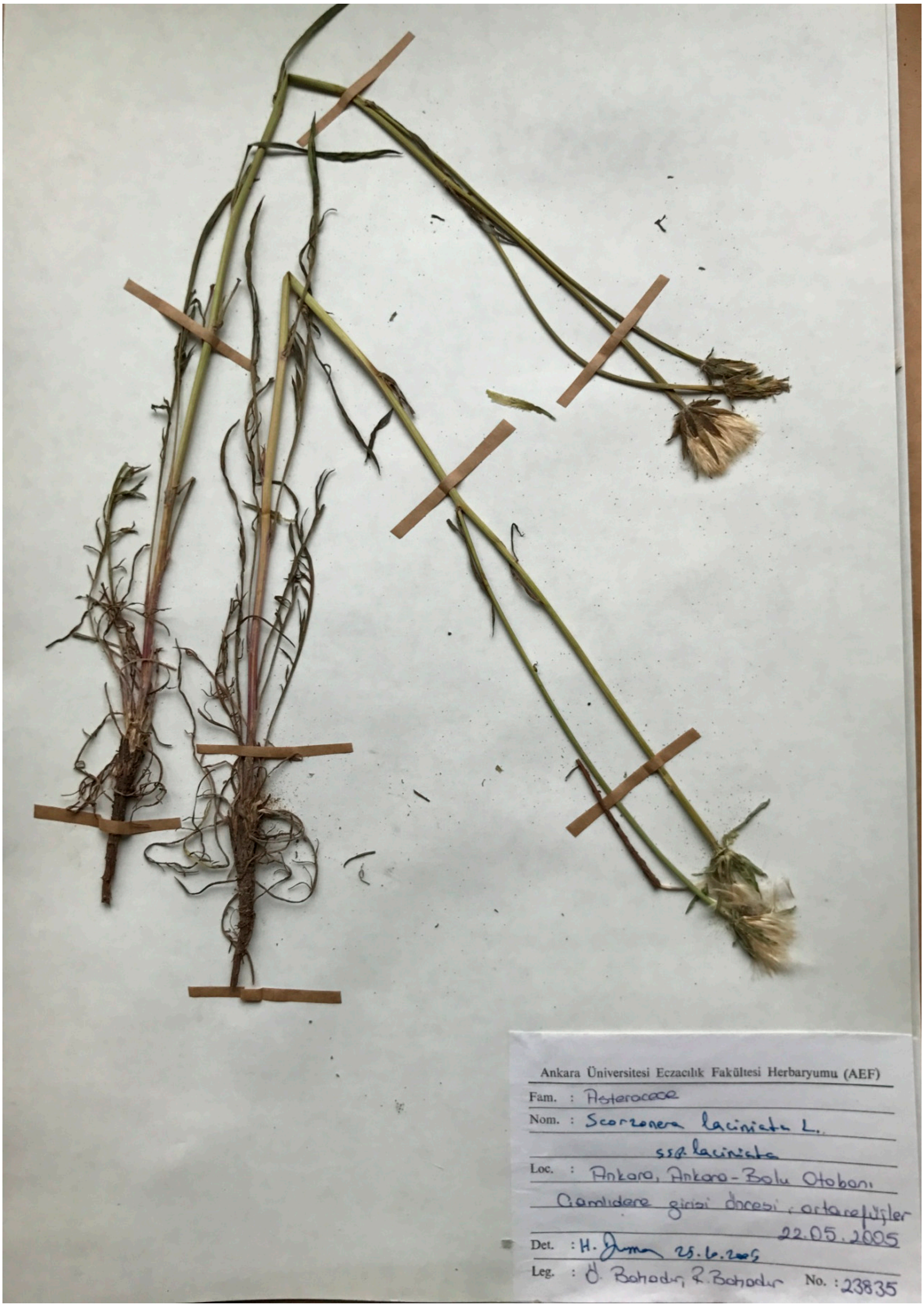

Figure S11: ***Scorzonera laciniata*** **L. ssp.** ***laciniata***

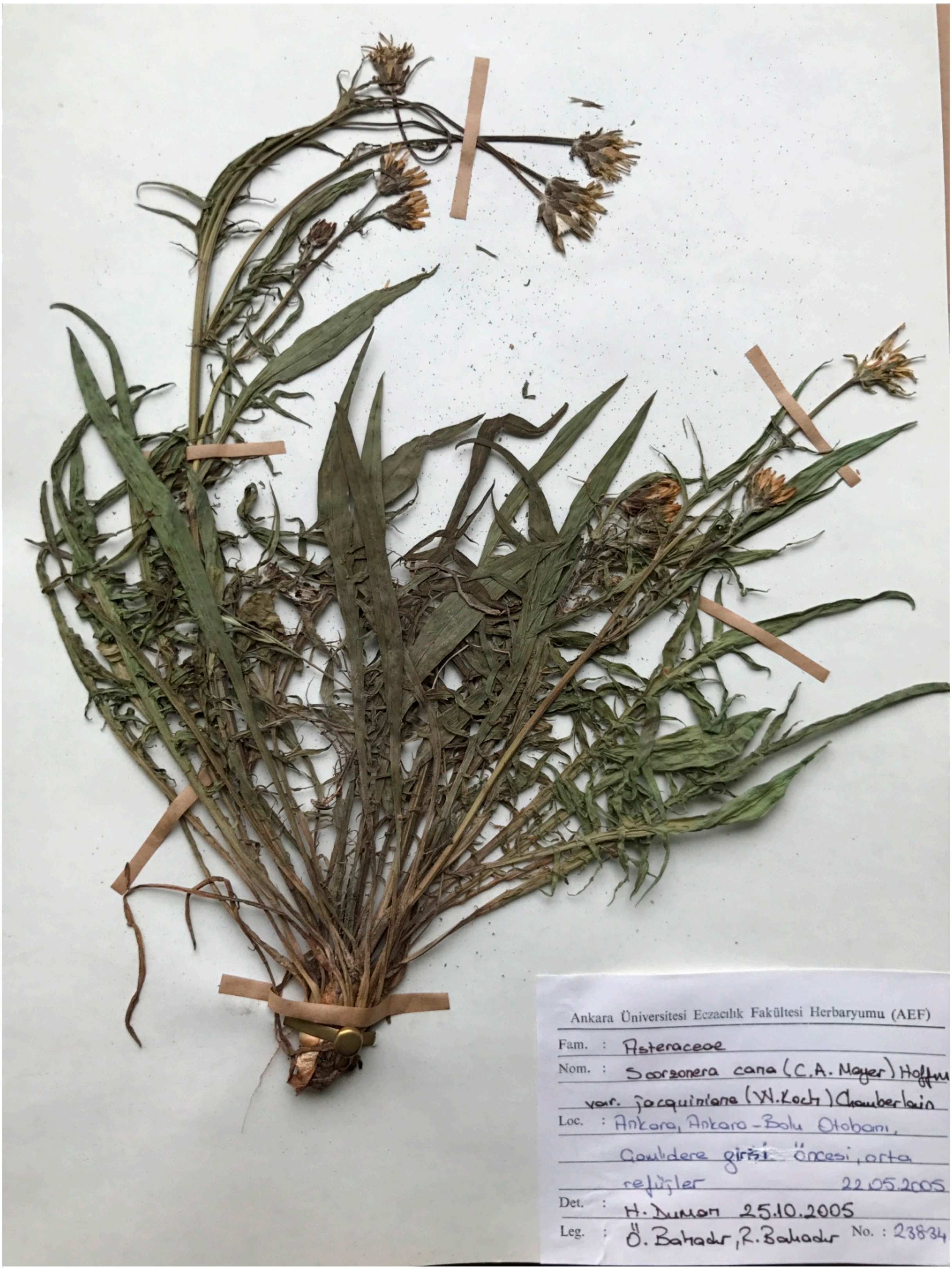

Figure S12: *Scorzonera cana* **(C.A. Meyer) Hoffm. var.** *jacquiniana* **(W. Koch) Chamberlain**

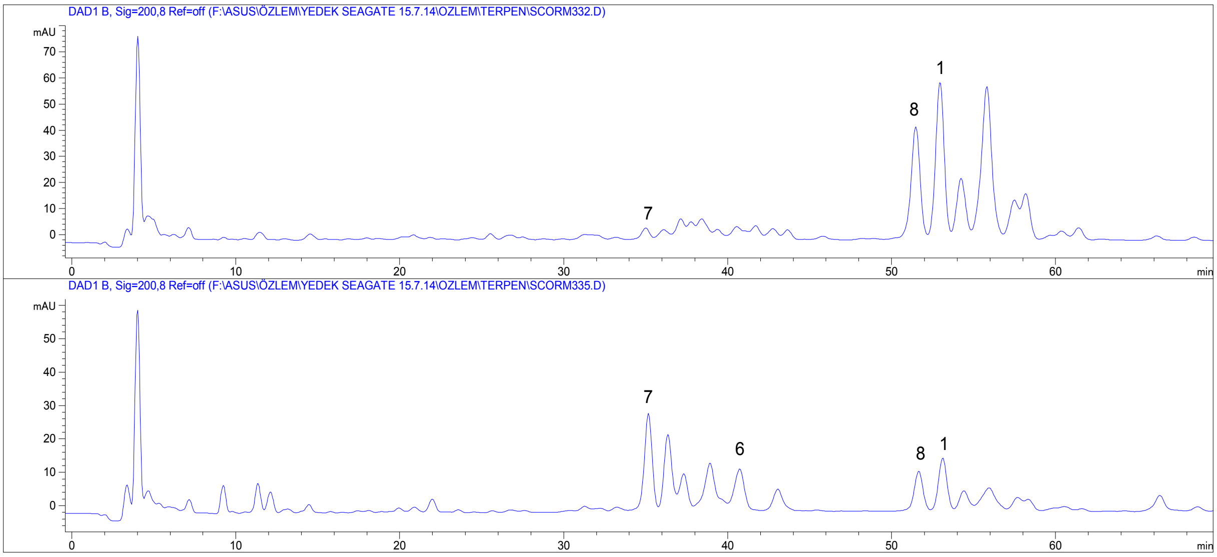

Figure S13: HPLC chromatograms of *S. latifolia* root (upper) and aerial (lower) part extract (λ 200 nm)

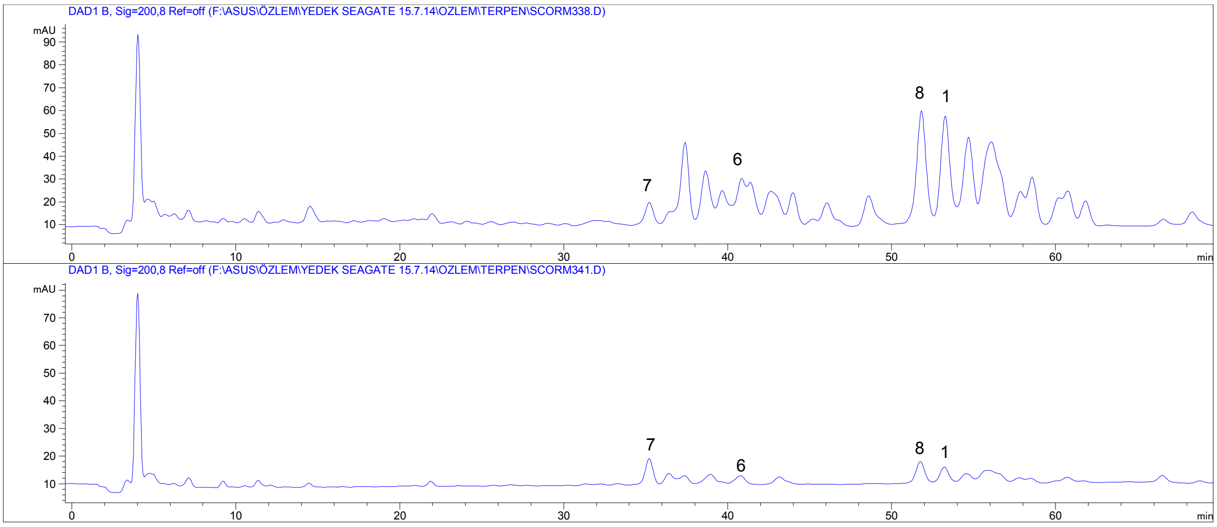

Figure S14: HPLC chromatograms of *S. tomentosa* root (upper) and aerial (lower) part extract (λ 200 nm)

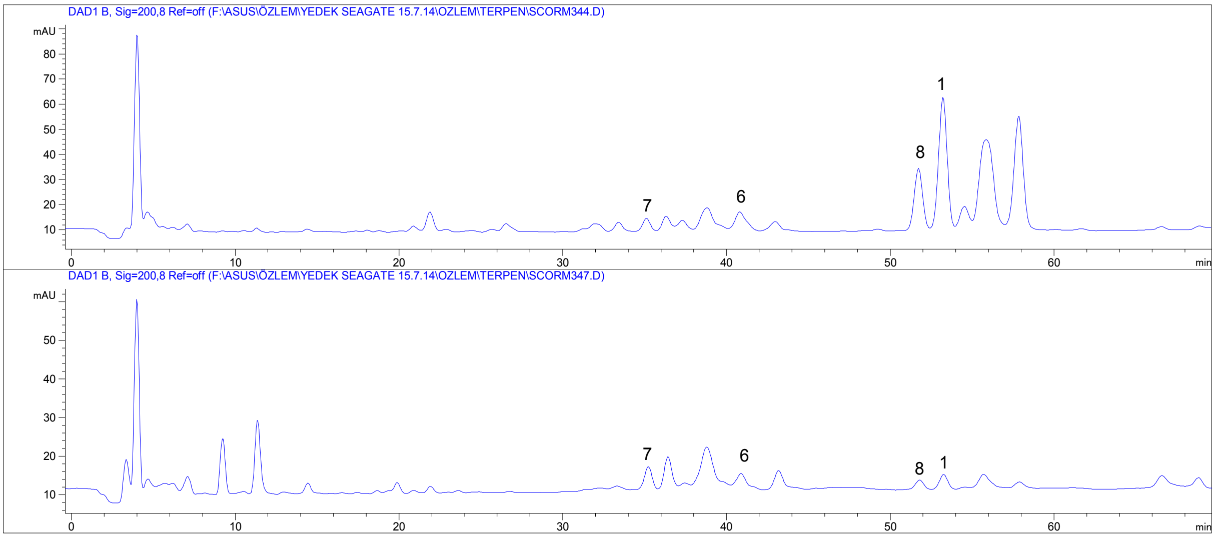

Figure S15: HPLC chromatograms of *S. mollis* ssp. *szowitsii* root (upper) and aerial (lower) part extract (λ 200 nm)

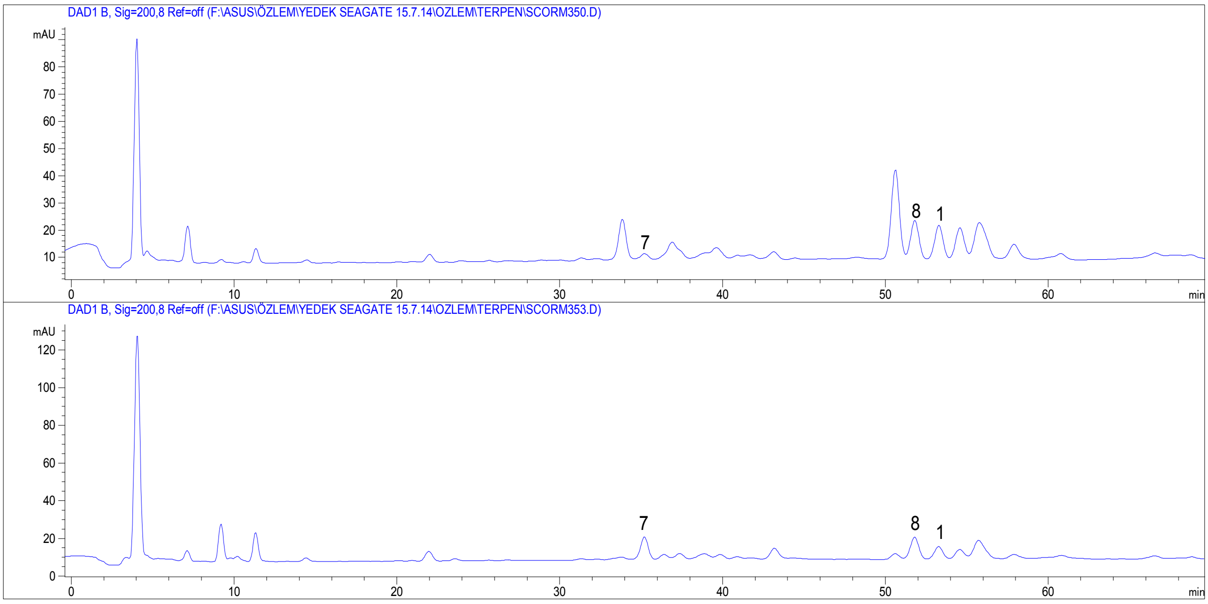

Figure S16: HPLC chromatograms of *S. parviflora* root (upper) and aerial (lower) part extract (λ 200 nm)

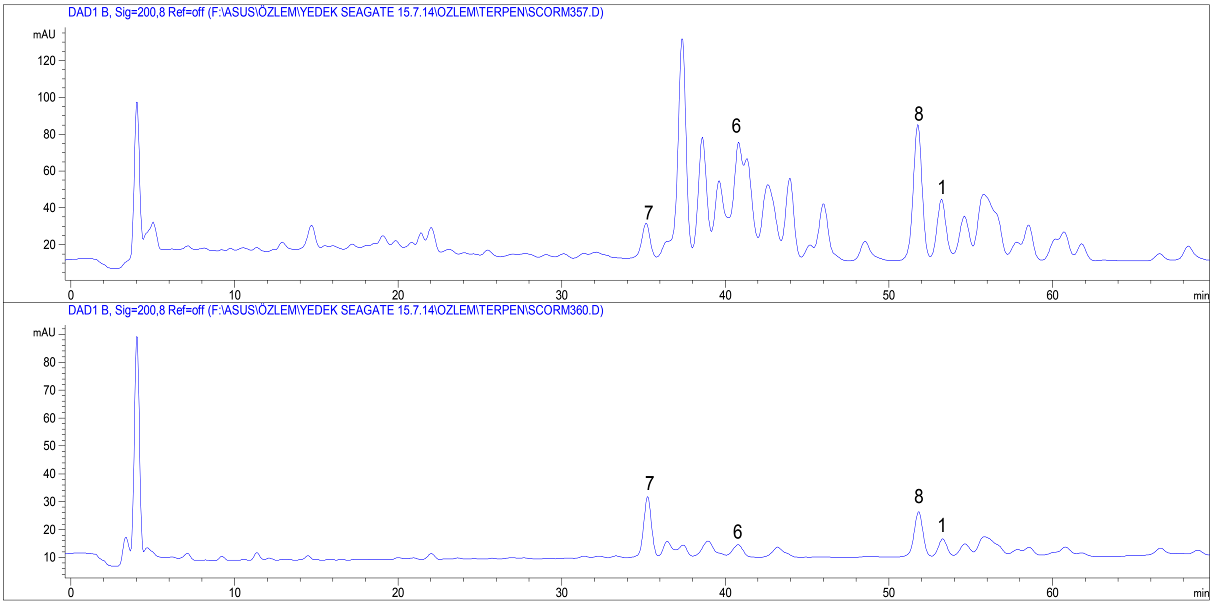

Figure S17: HPLC chromatograms of *S. cinerea* root (upper) and aerial (lower) part extract (λ 200 nm)

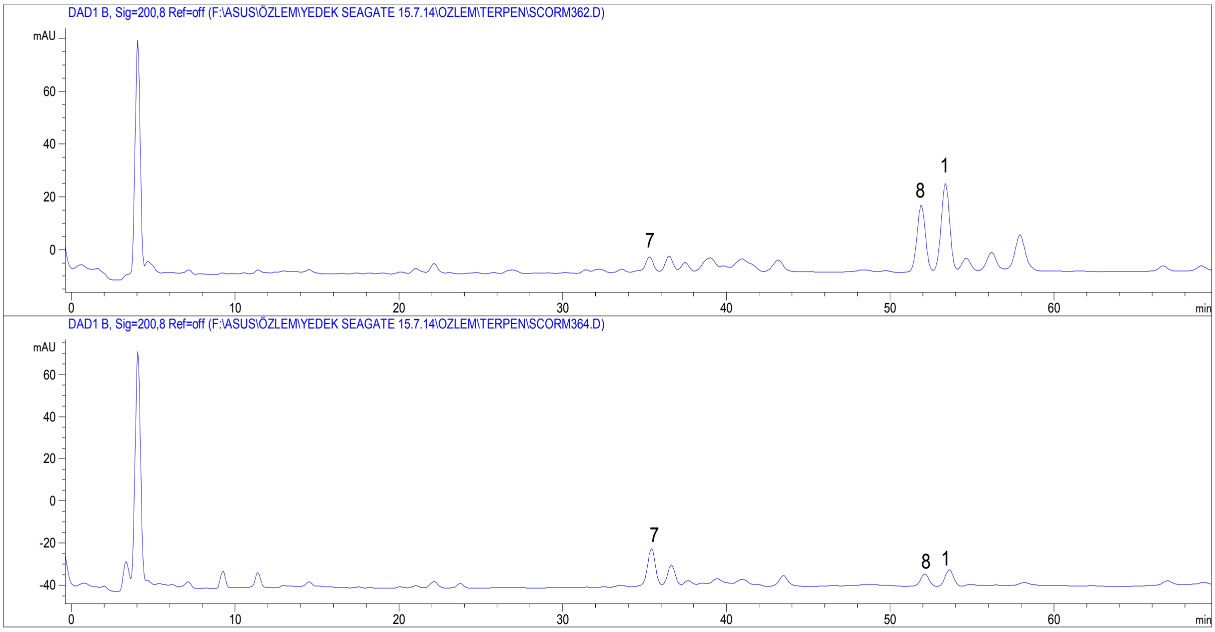

Figure S18: HPLC chromatograms of *S. suberosa* ssp. *suberosa* root (upper) and aerial (lower) part extract (λ 200 nm)

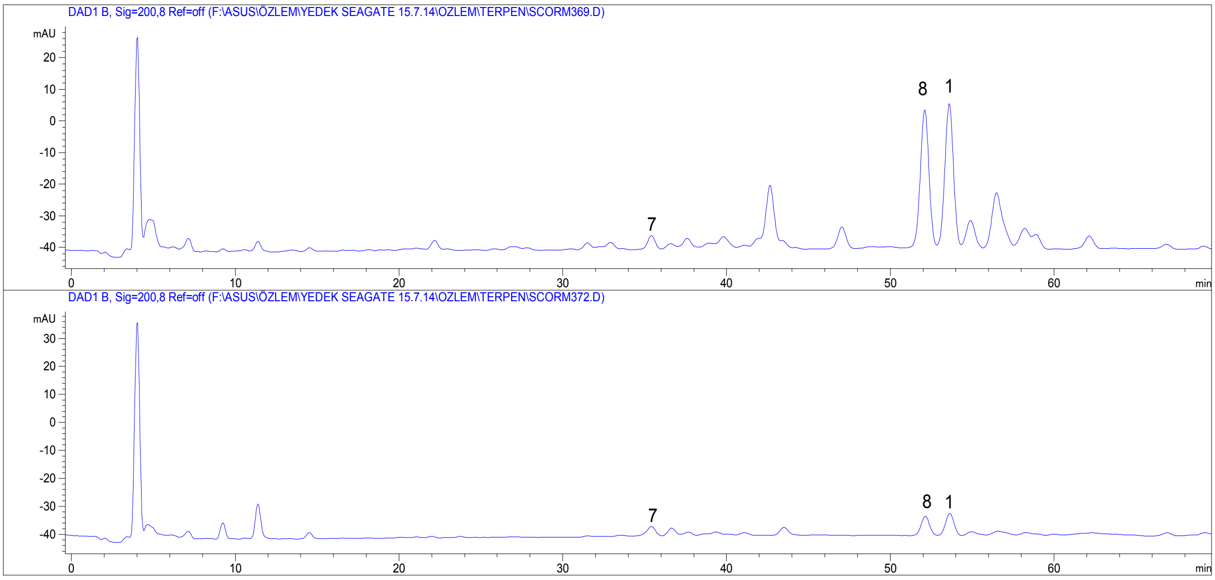

Figure S19: HPLC chromatograms of *S. eriophora* root (upper) and aerial (lower) part extract (λ 200 nm)

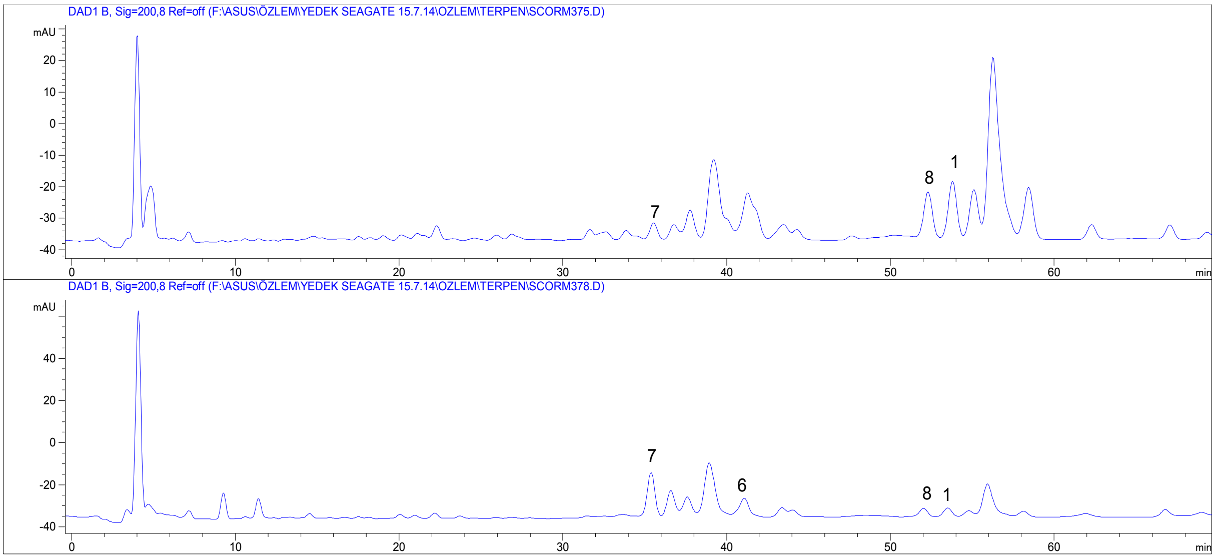

Figure S20: HPLC chromatograms of *S. incisa* root (upper) and aerial (lower) part extract (λ 200 nm)

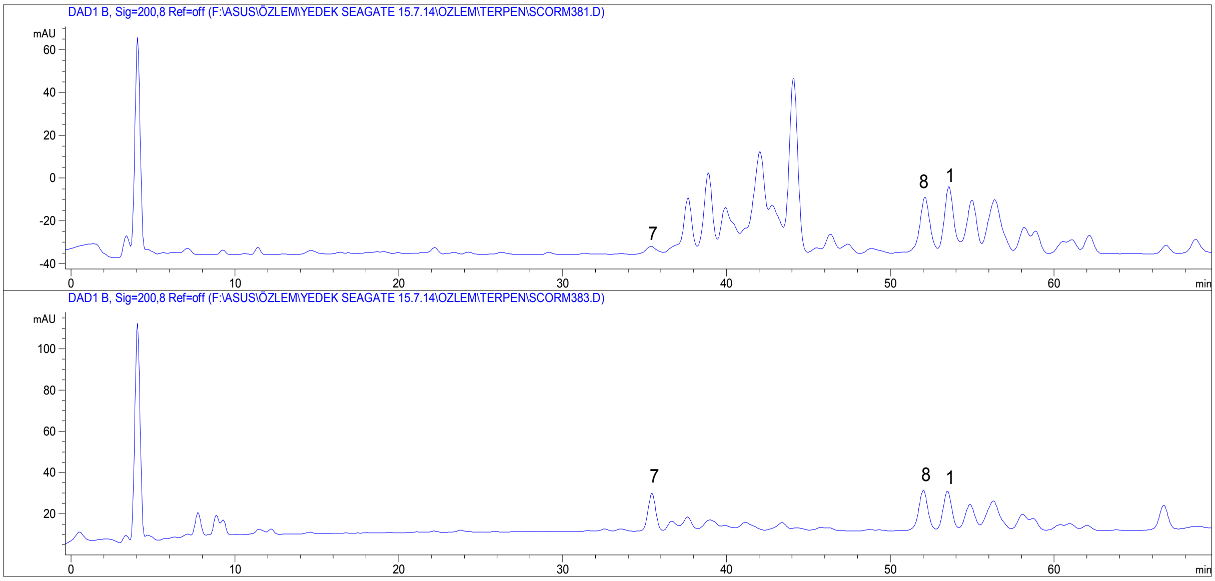

Figure S21: HPLC chromatograms of *S. mirabilis* root (upper) and aerial (lower) part extract (λ 200 nm)

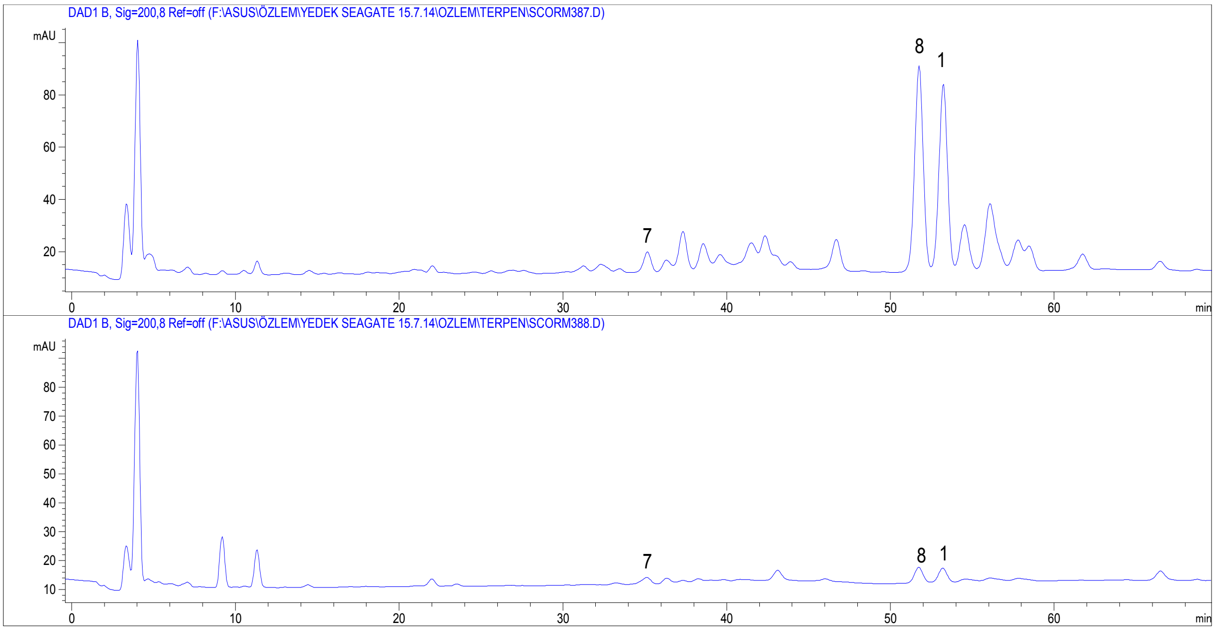

Figure S22: HPLC chromatograms *of S. sublanata* root (upper) and aerial (lower) part extract (λ 200 nm)

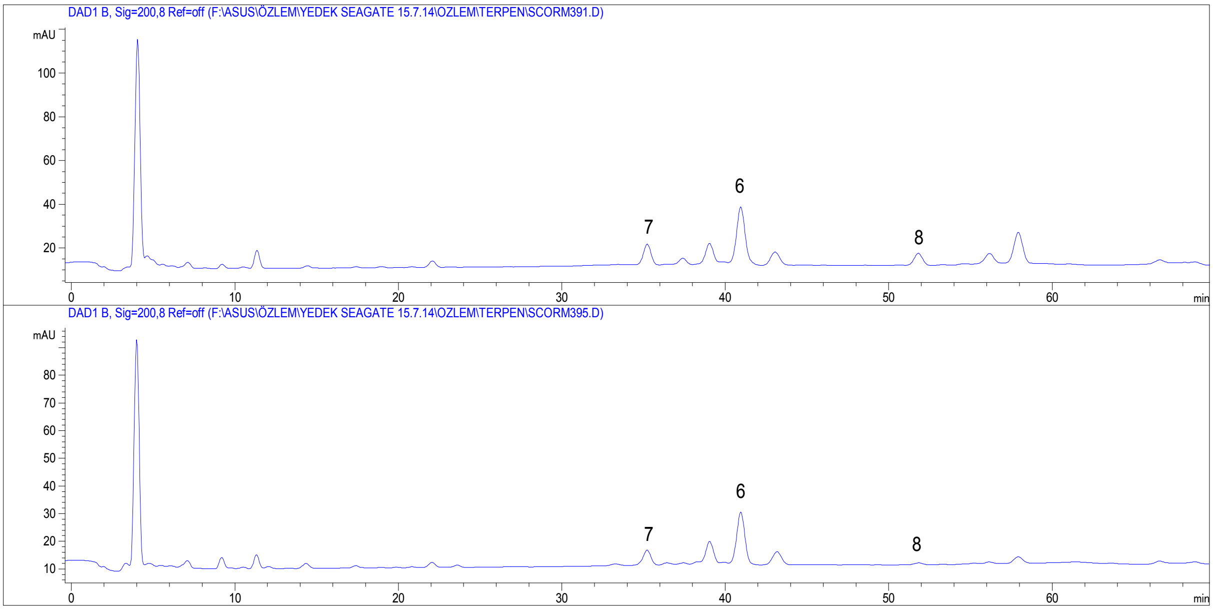

Figure S23: HPLC chromatograms of *S. acuminata* root (upper) and aerial (lower) part extract (λ 200 nm)

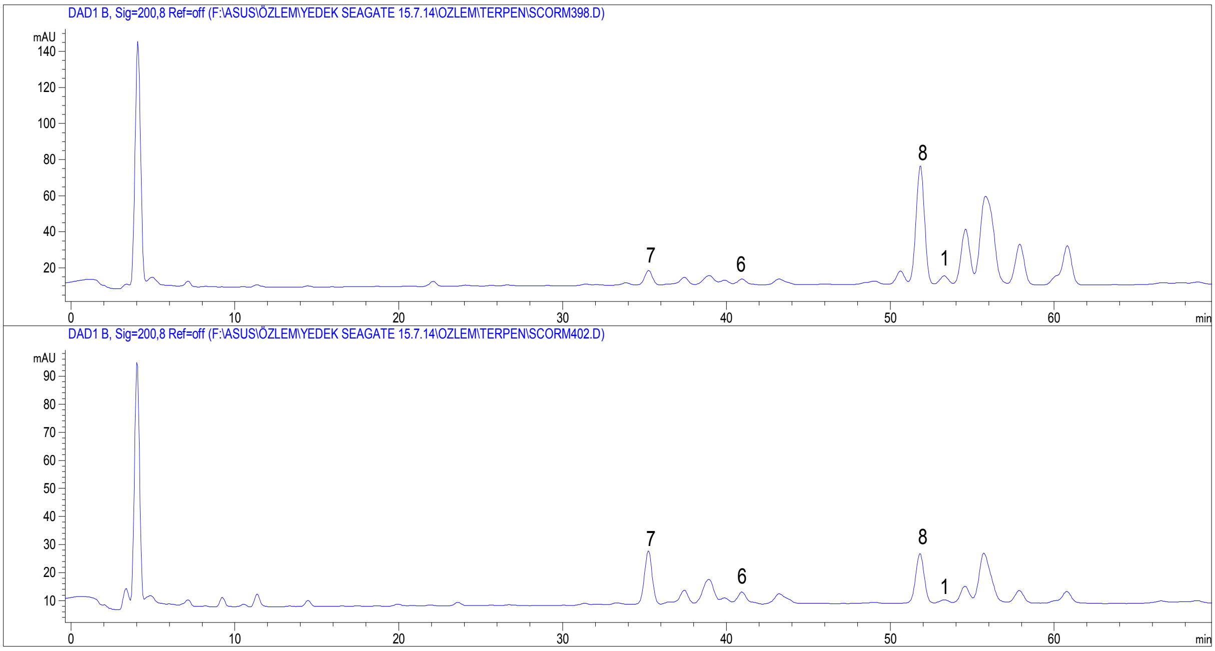

Figure S24: HPLC chromatograms of *S. laciniata* ssp. *laciniata* root (upper) and aerial (lower) part extract (λ 200 nm)

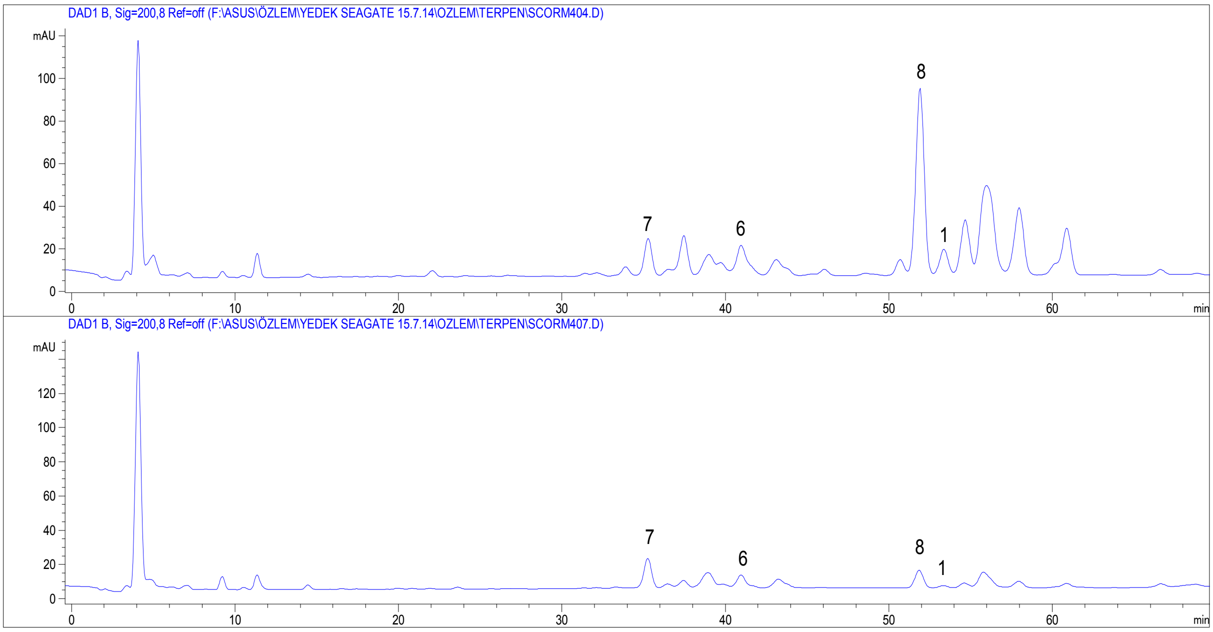

Figure S25: HPLC chromatograms of *S. cana* var. *jacquiniana* root (upper) and aerial (lower) part extract (λ 200 nm)

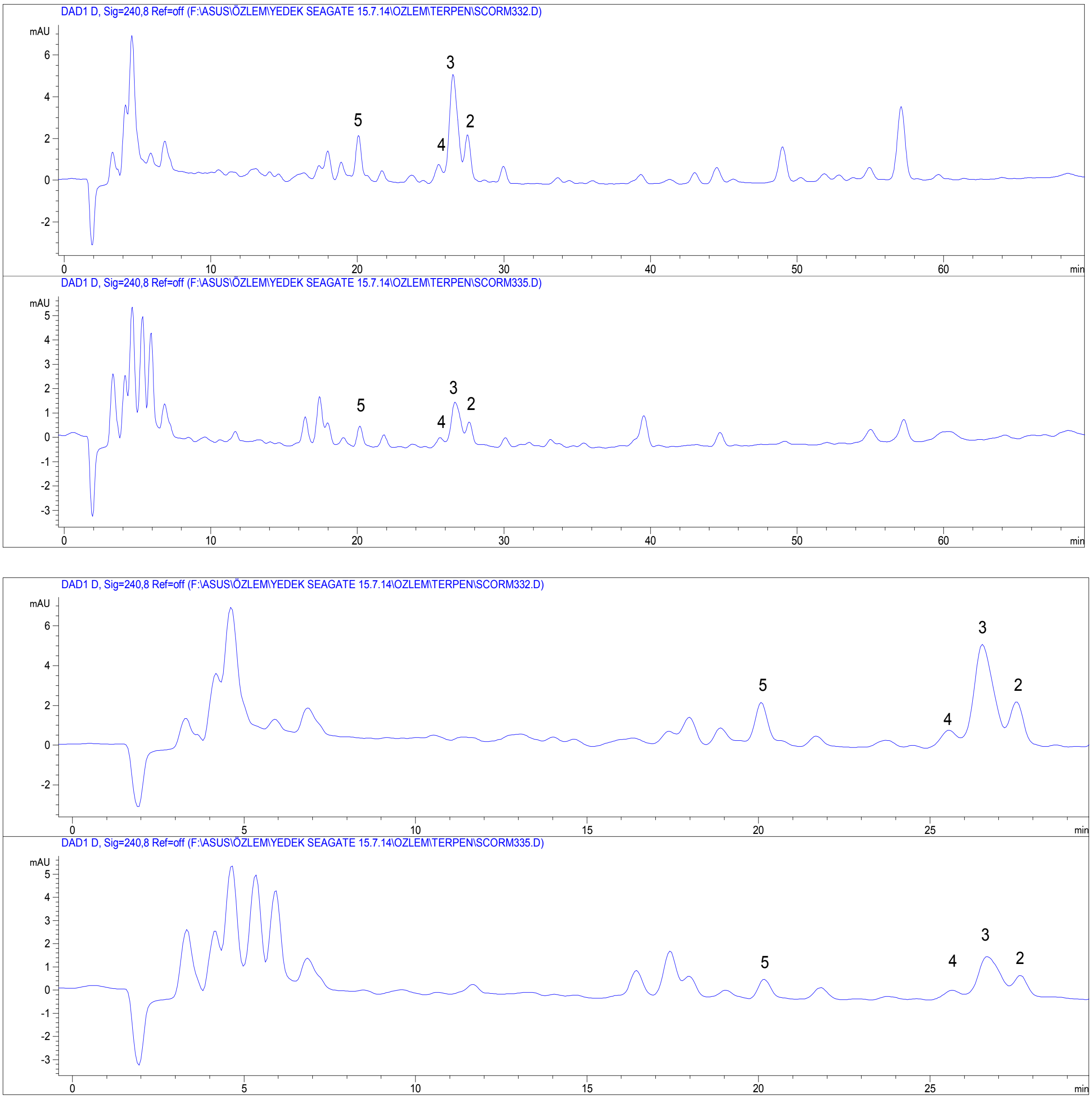

Figure S26: HPLC chromatograms of *S. latifolia* root (upper) and aerial (lower) part extract (λ 200 and 240 nm)

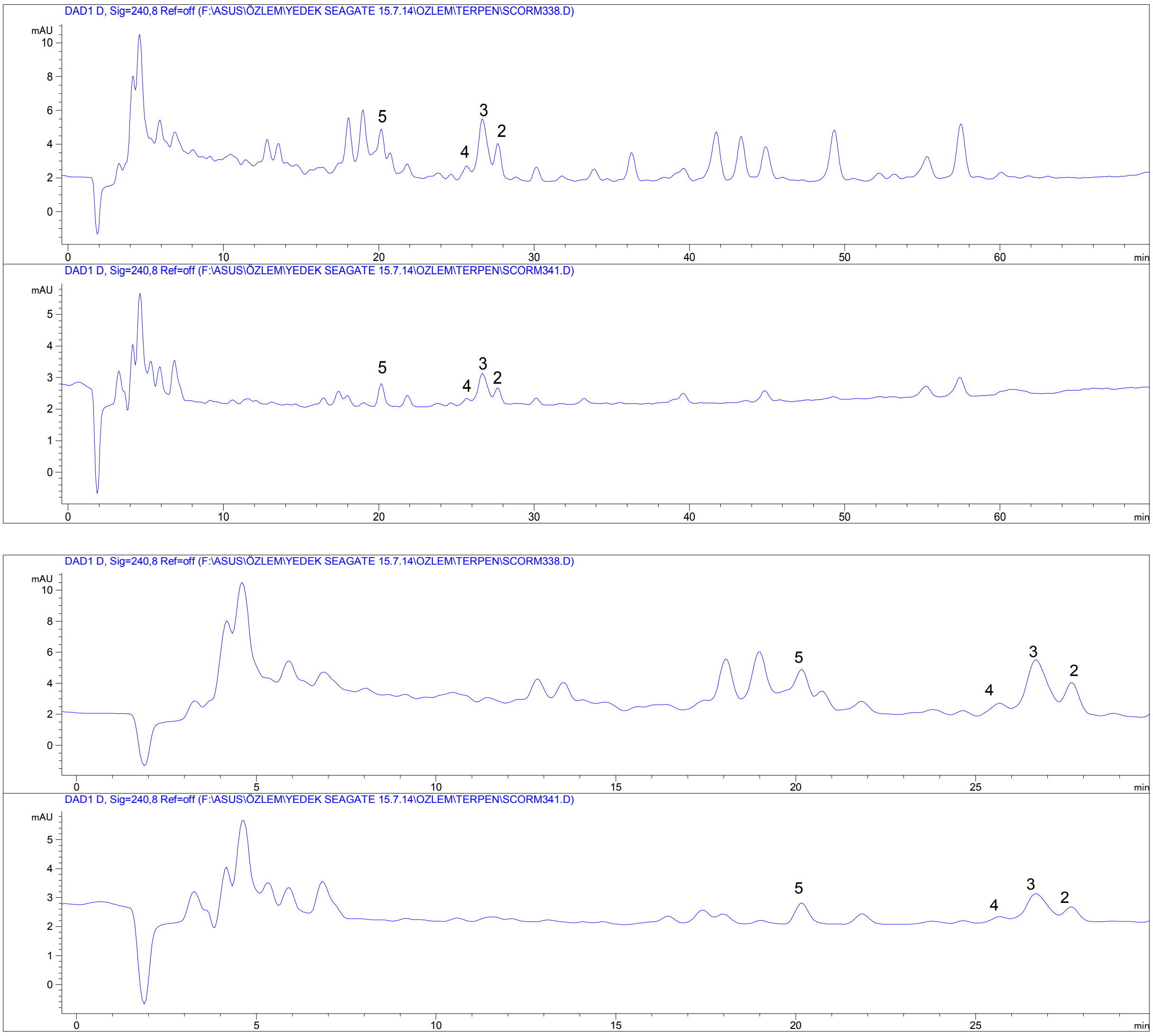

Figure S27: HPLC chromatograms of *S. tomentosa* root (upper) and aerial (lower) part extract (λ 200 and 240 nm)

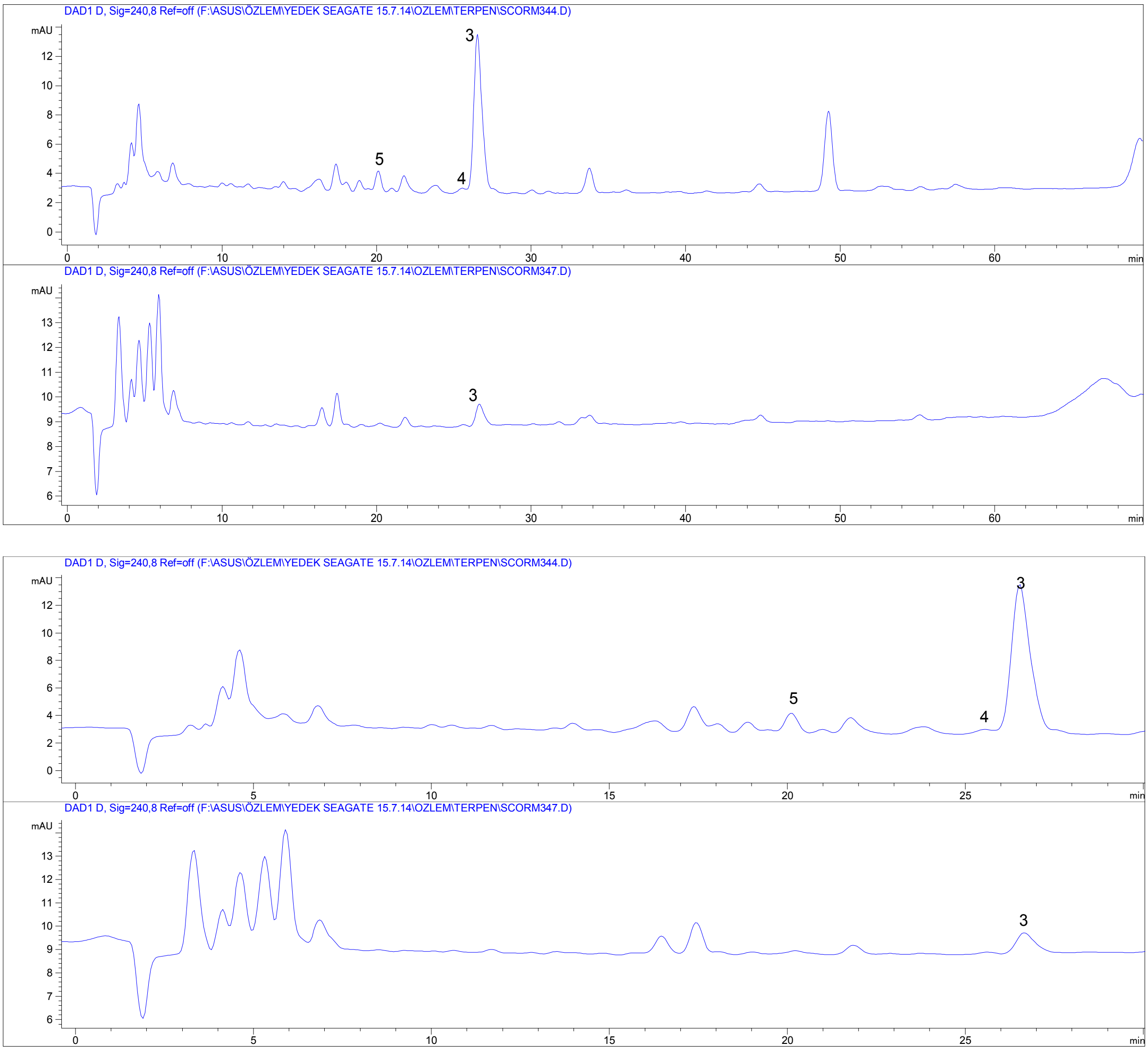

Figure S28: HPLC chromatograms of *S. mollis* ssp. *szowitsii* root (upper) and aerial (lower) part extract (λ 200 and 240 nm)

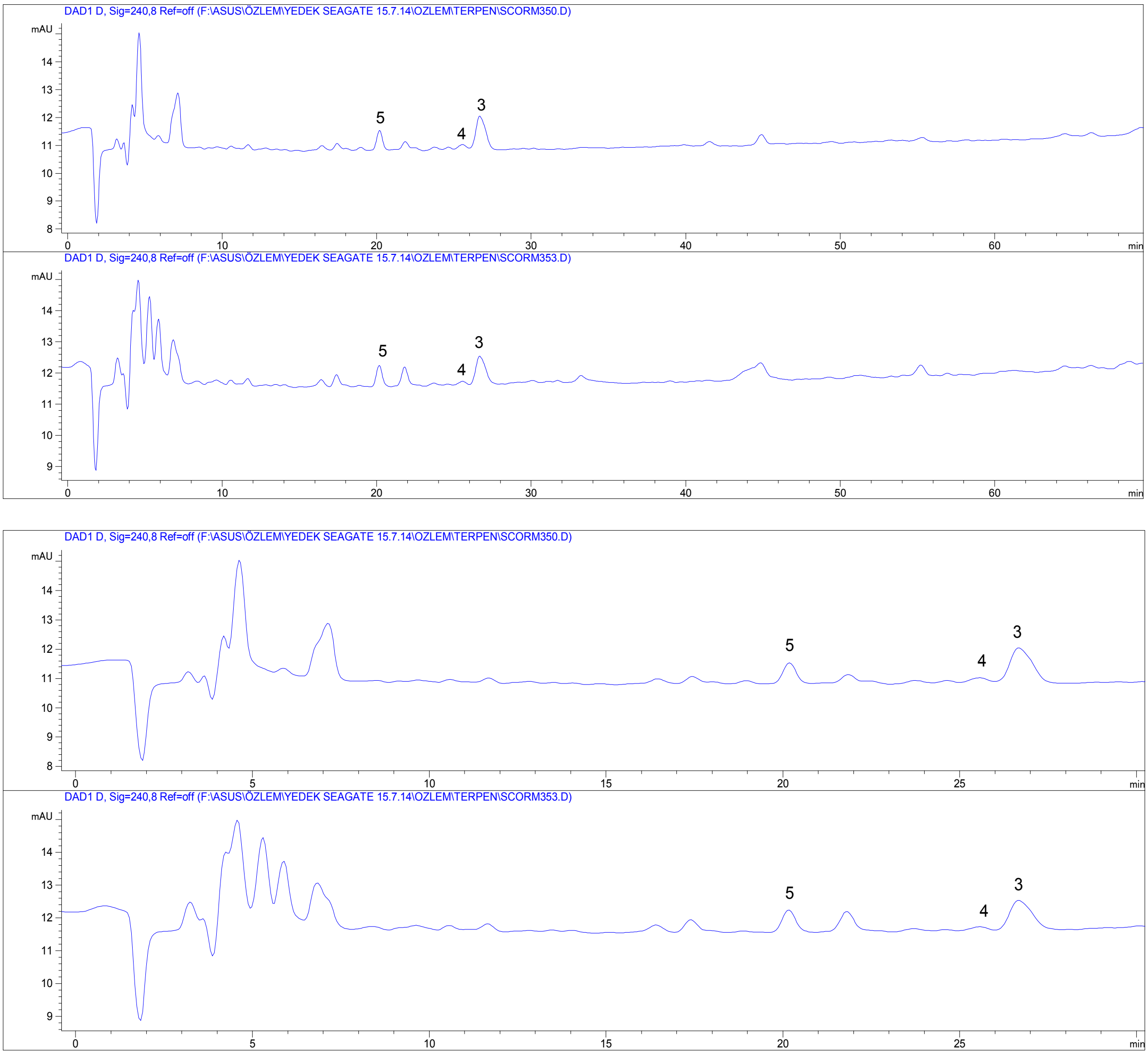

Figure S29: HPLC chromatograms of *S.parviflora* root (upper) and aerial (lower) part extract (λ 200 and 240 nm)

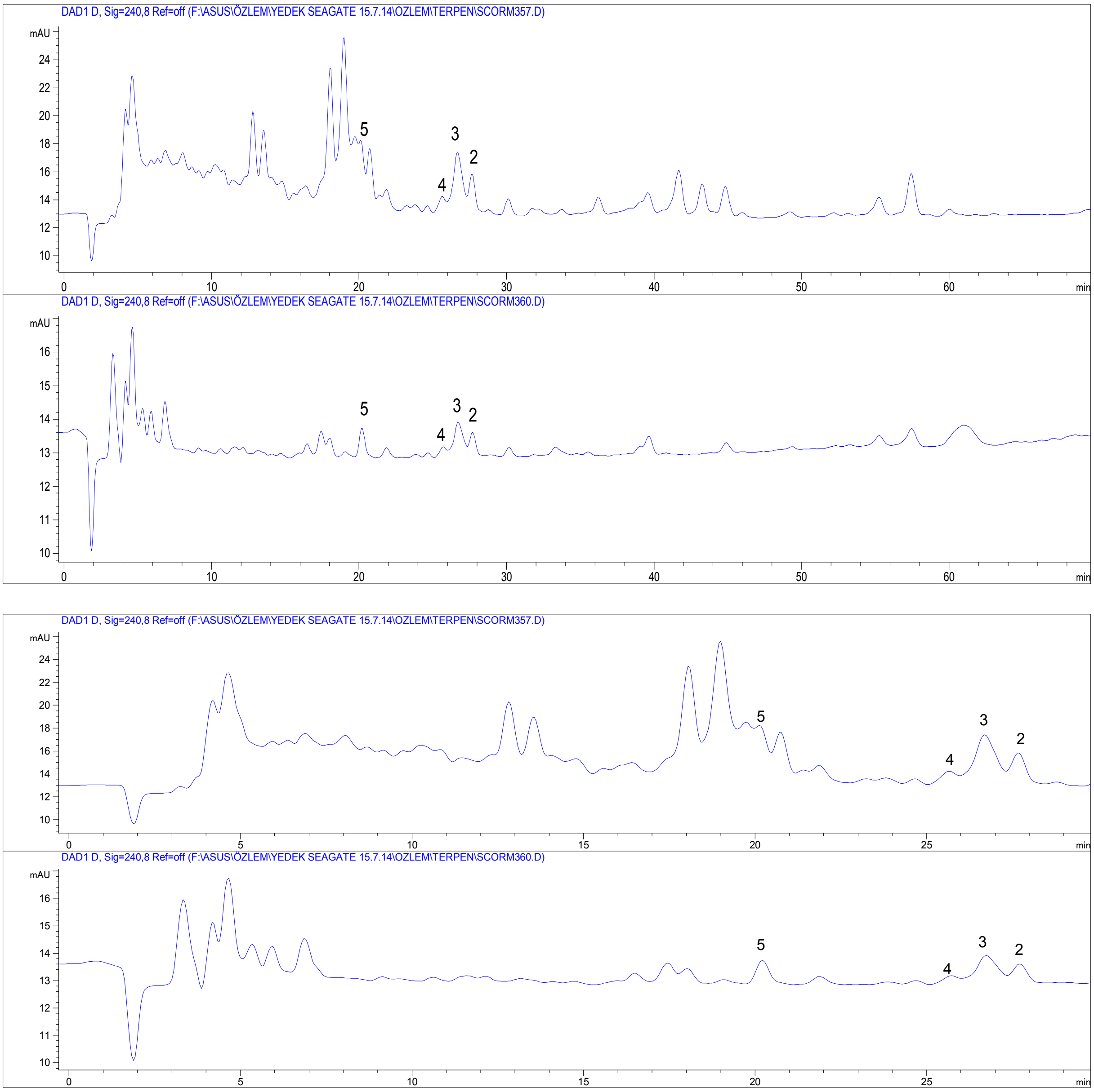

Figure S30: HPLC chromatograms of *S. cinerea* root (upper) and aerial (lower) part extract (λ 200 and 240 nm)

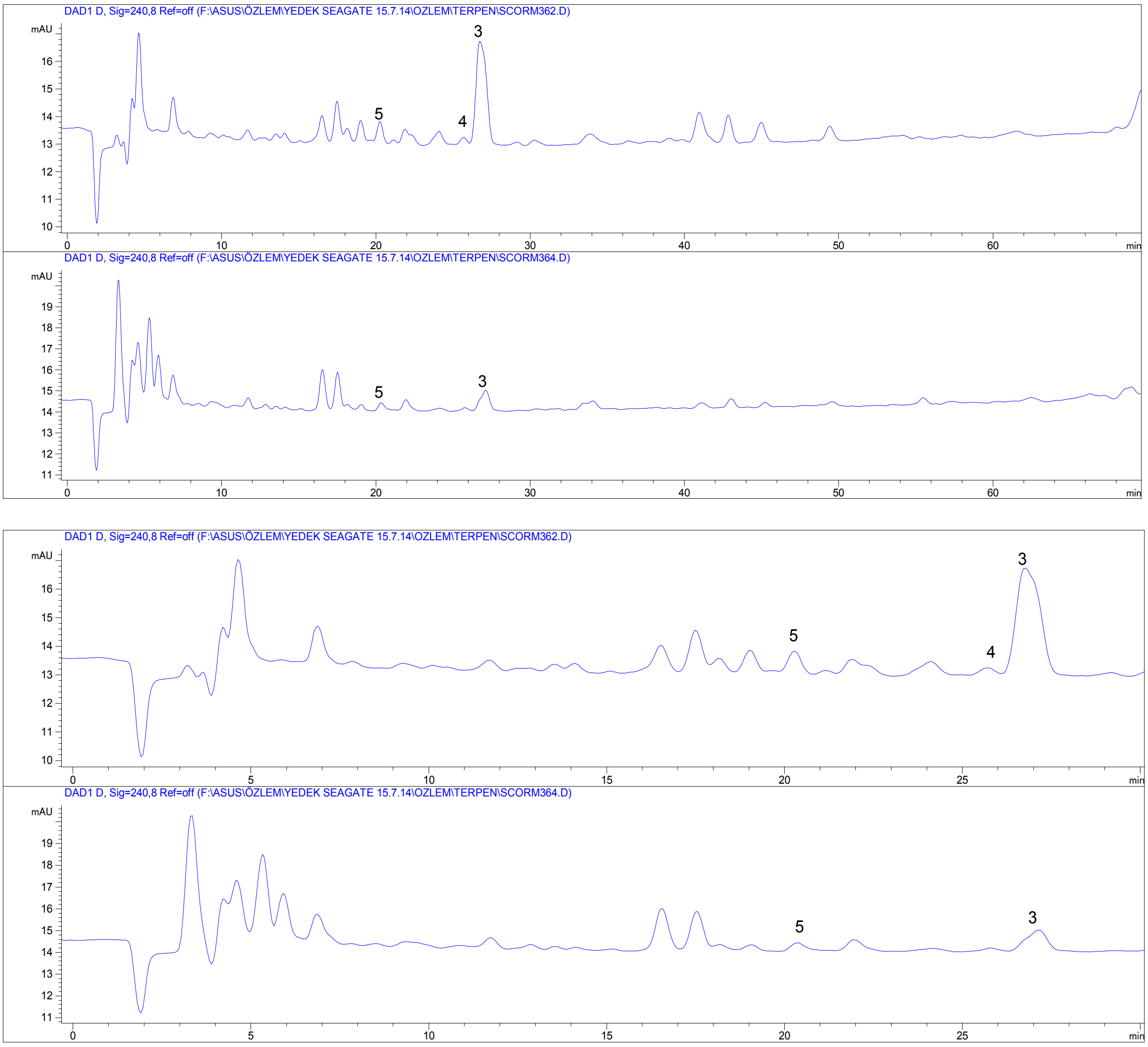

Figure S31: HPLC chromatograms of *S. suberosa* ssp. *suberosa* (upper) and aerial (lower) part extract (λ 200 and 240 nm)

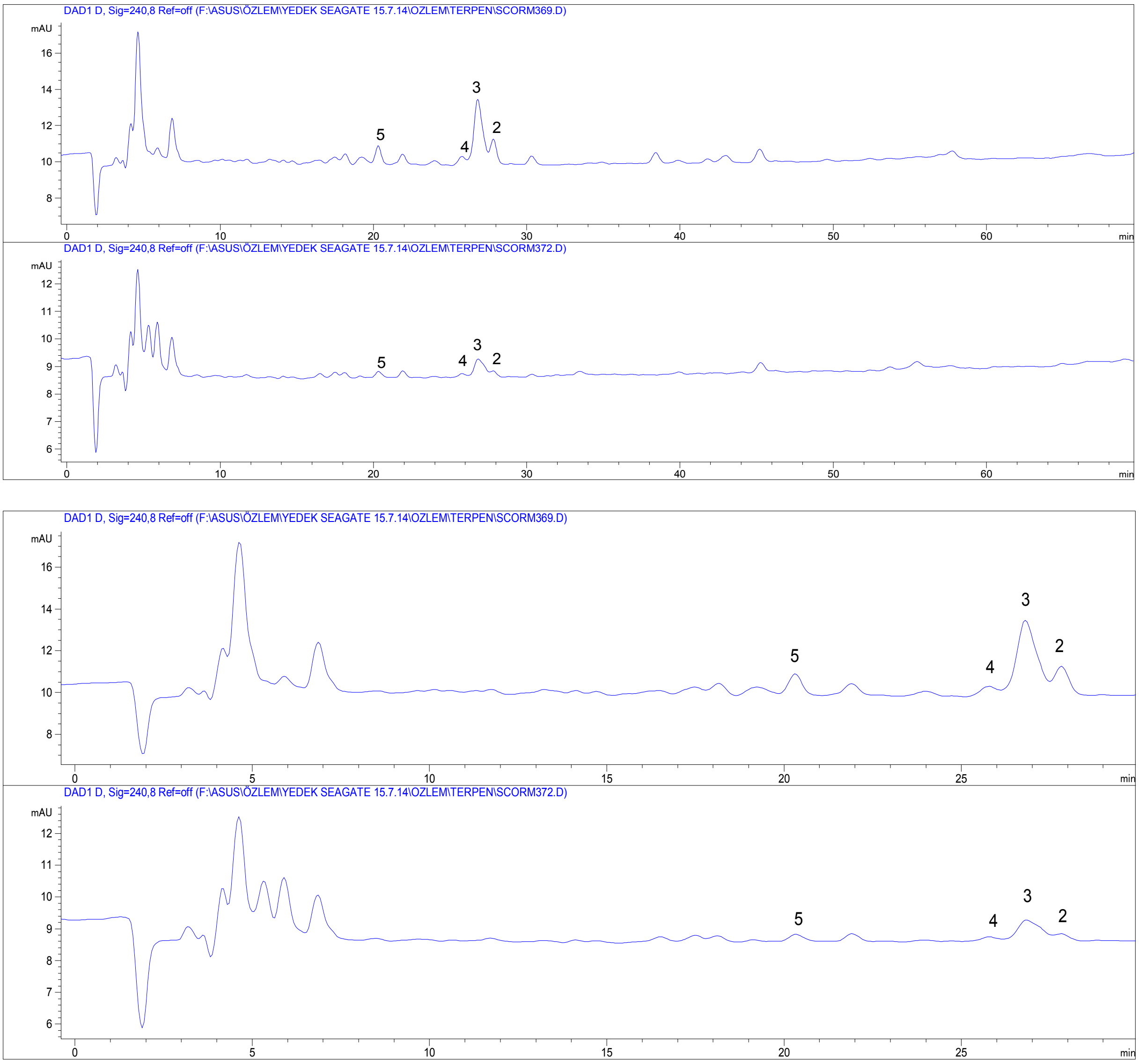

Figure S32: HPLC chromatograms of *S. eriophora* root (upper) and aerial (lower) part extract (λ 200 and 240 nm)

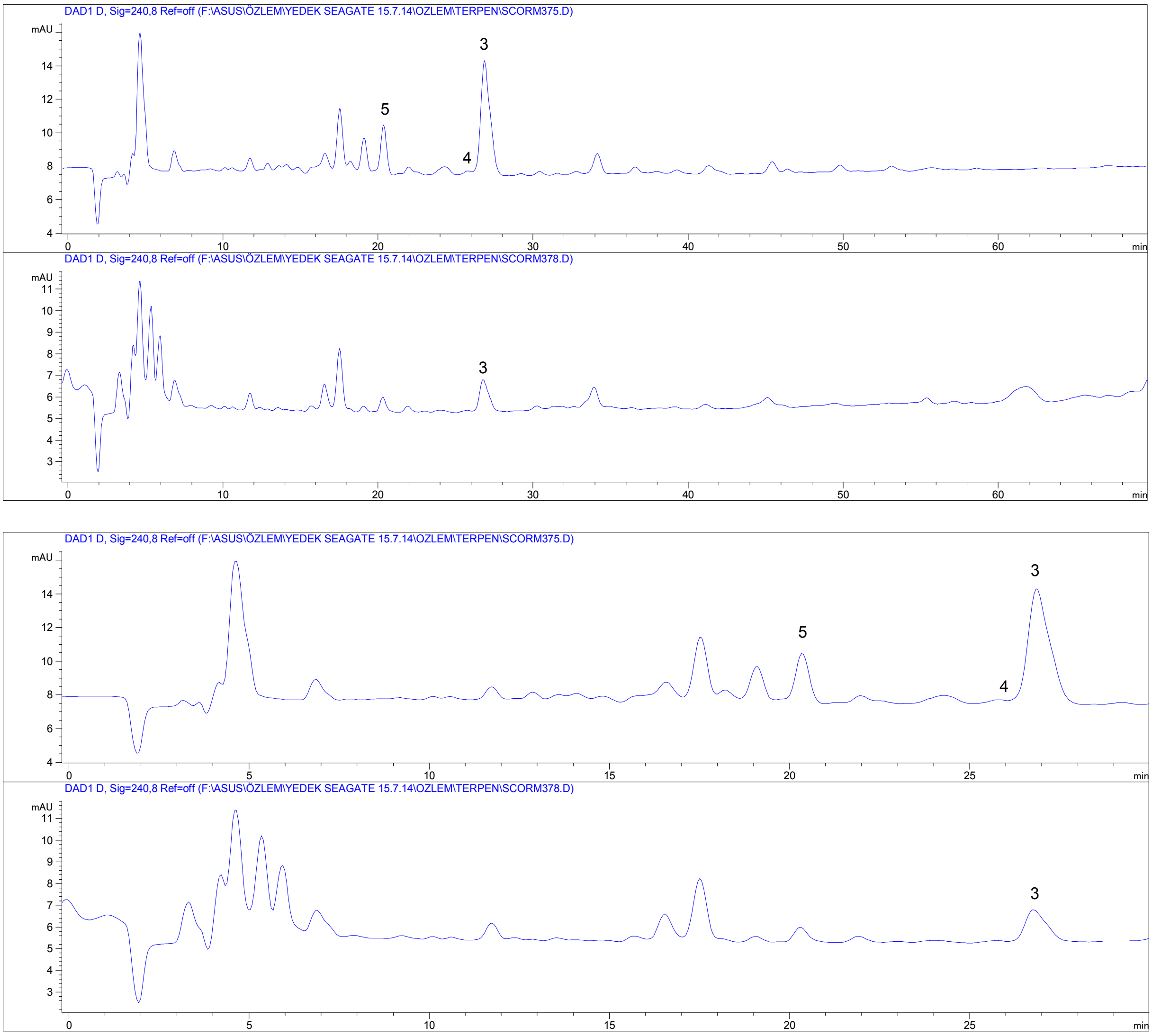

Figure S33: HPLC chromatograms of *S. incisa* root (upper) and aerial (lower) part extract (λ 200 and 240 nm)

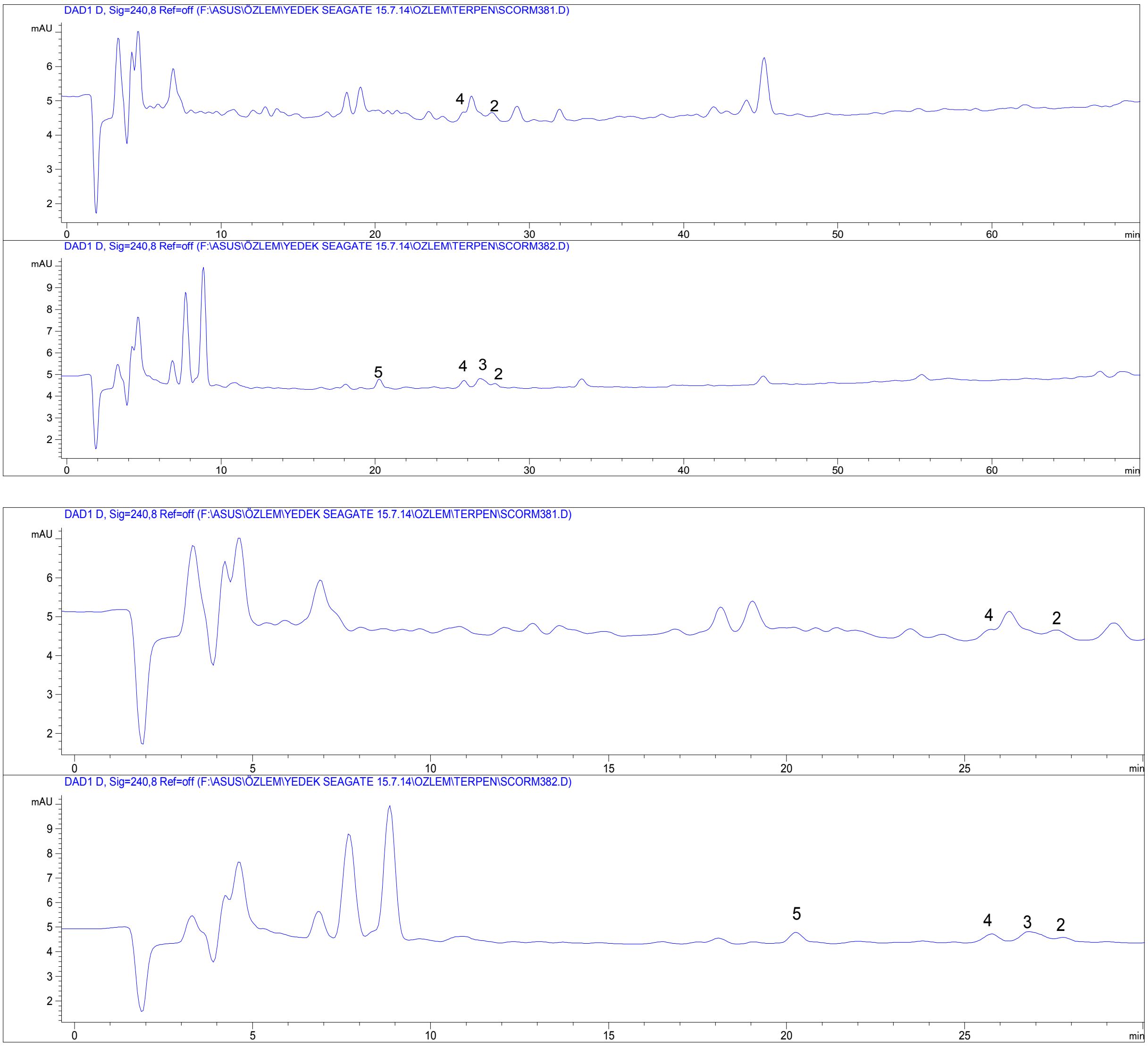

Figure S34: HPLC chromatograms of *S. mirabilis* root (upper) and aerial (lower) part extract (λ 200 and 240 nm)

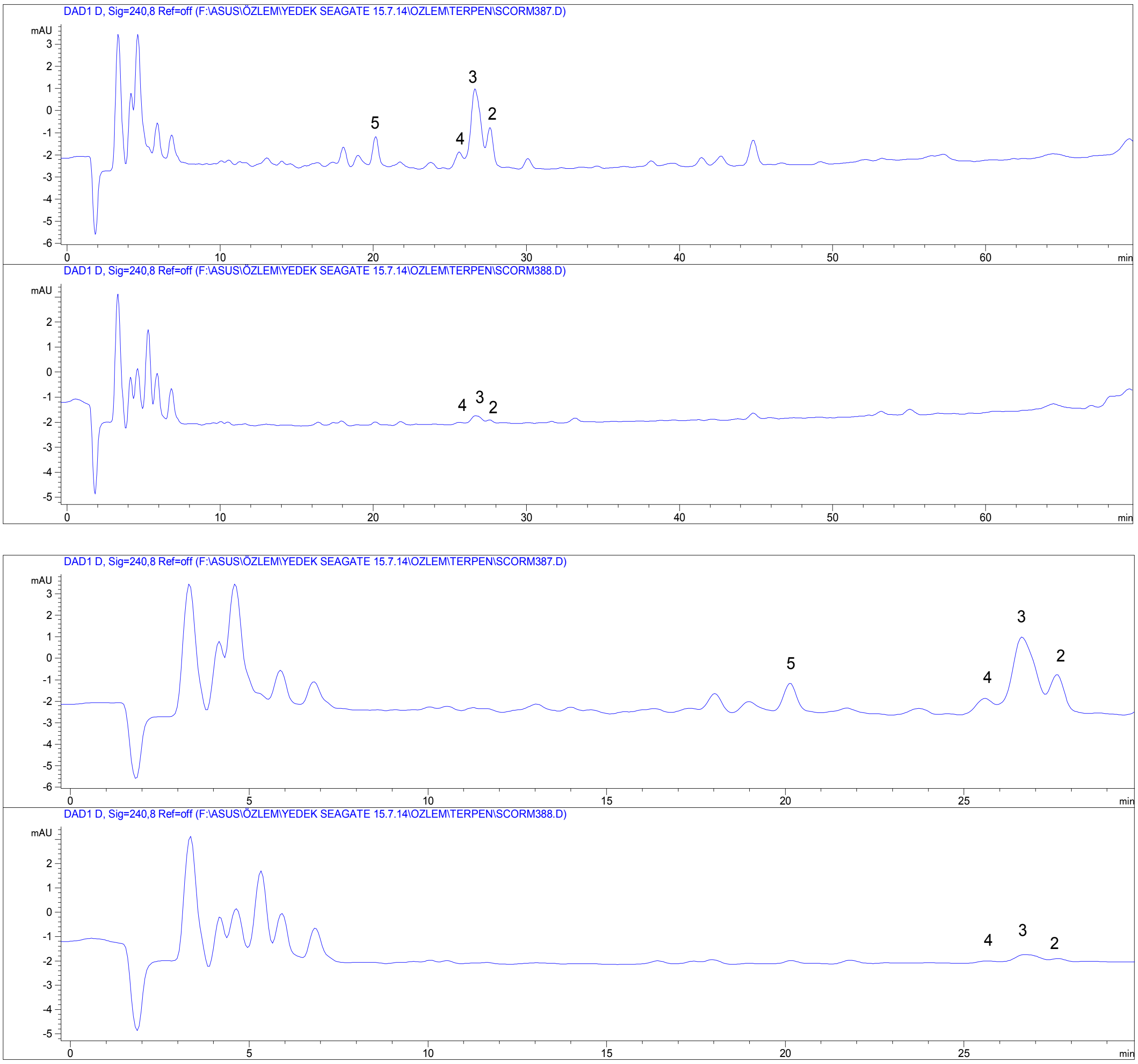

Figure S35: HPLC chromatograms of *S. sublanata* root (upper) and aerial (lower) part extract (λ 200 and 240 nm)

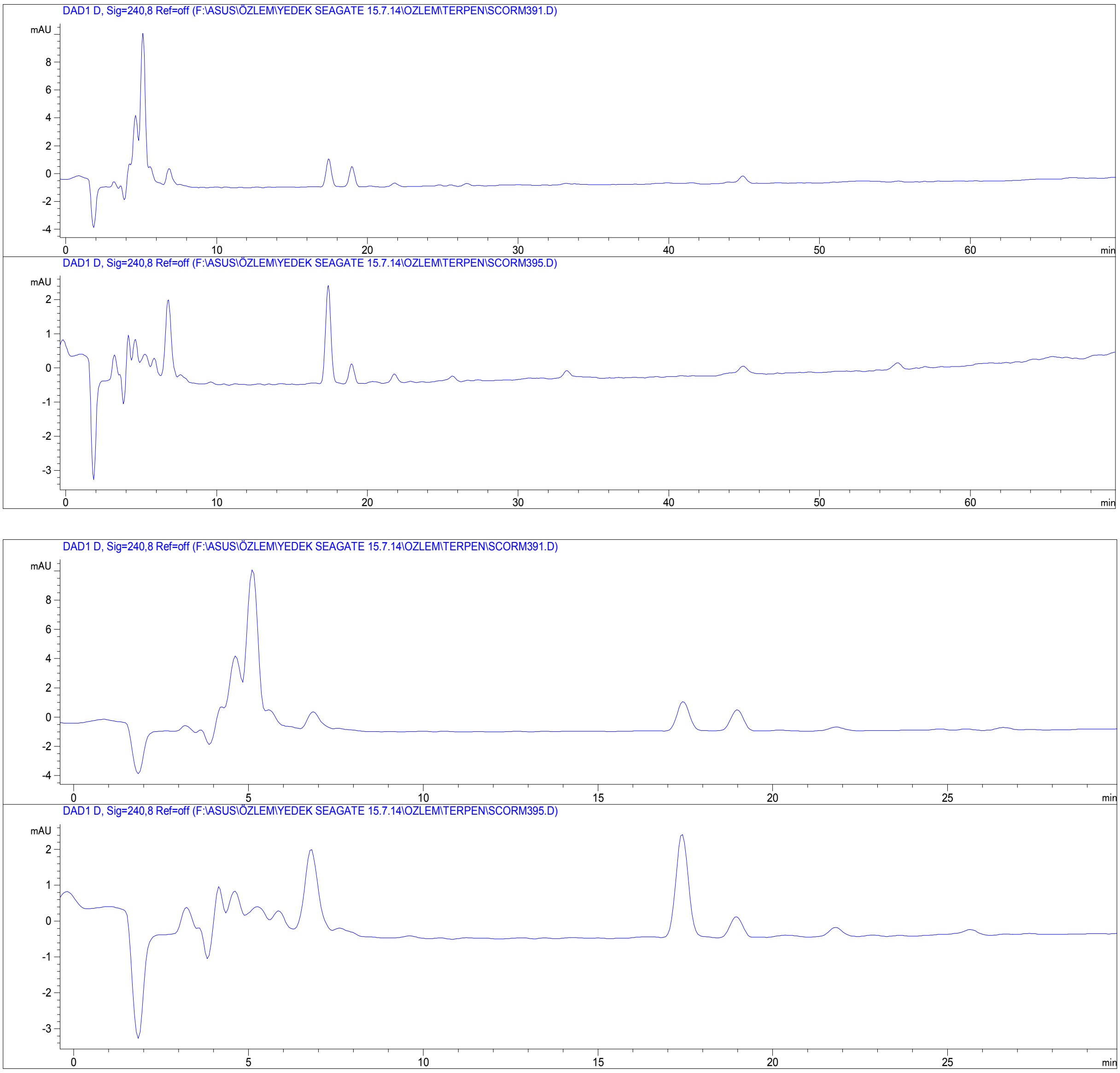

Figure S36: HPLC chromatograms of *S. acuminata* root (upper) and aerial (lower) part extract (λ 200 and 240 nm)

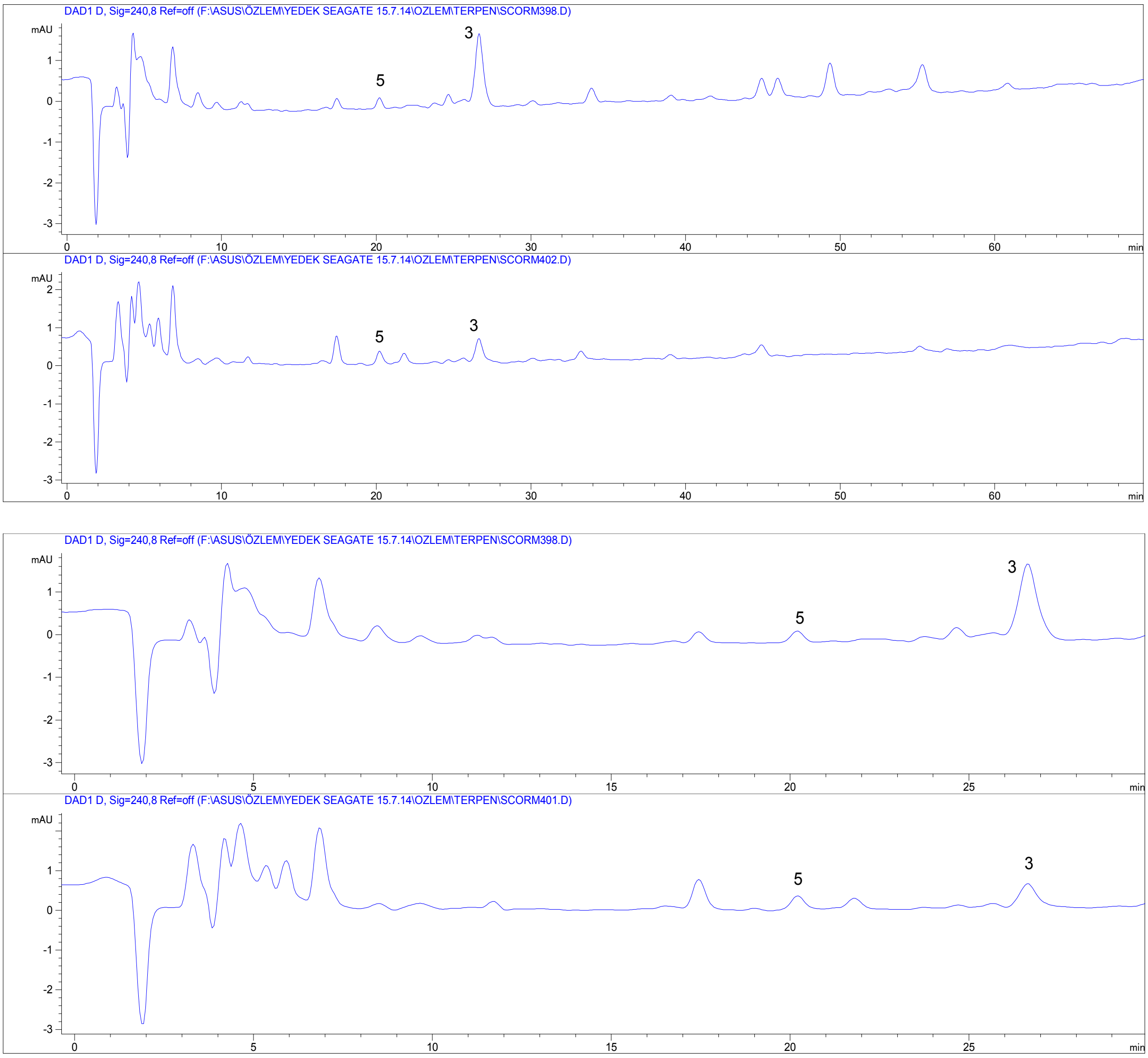

Figure S37: HPLC chromatograms of *S. laciniata* ssp. *laciniata* root (upper) and aerial (lower) part extract (λ 200 and 240 nm)

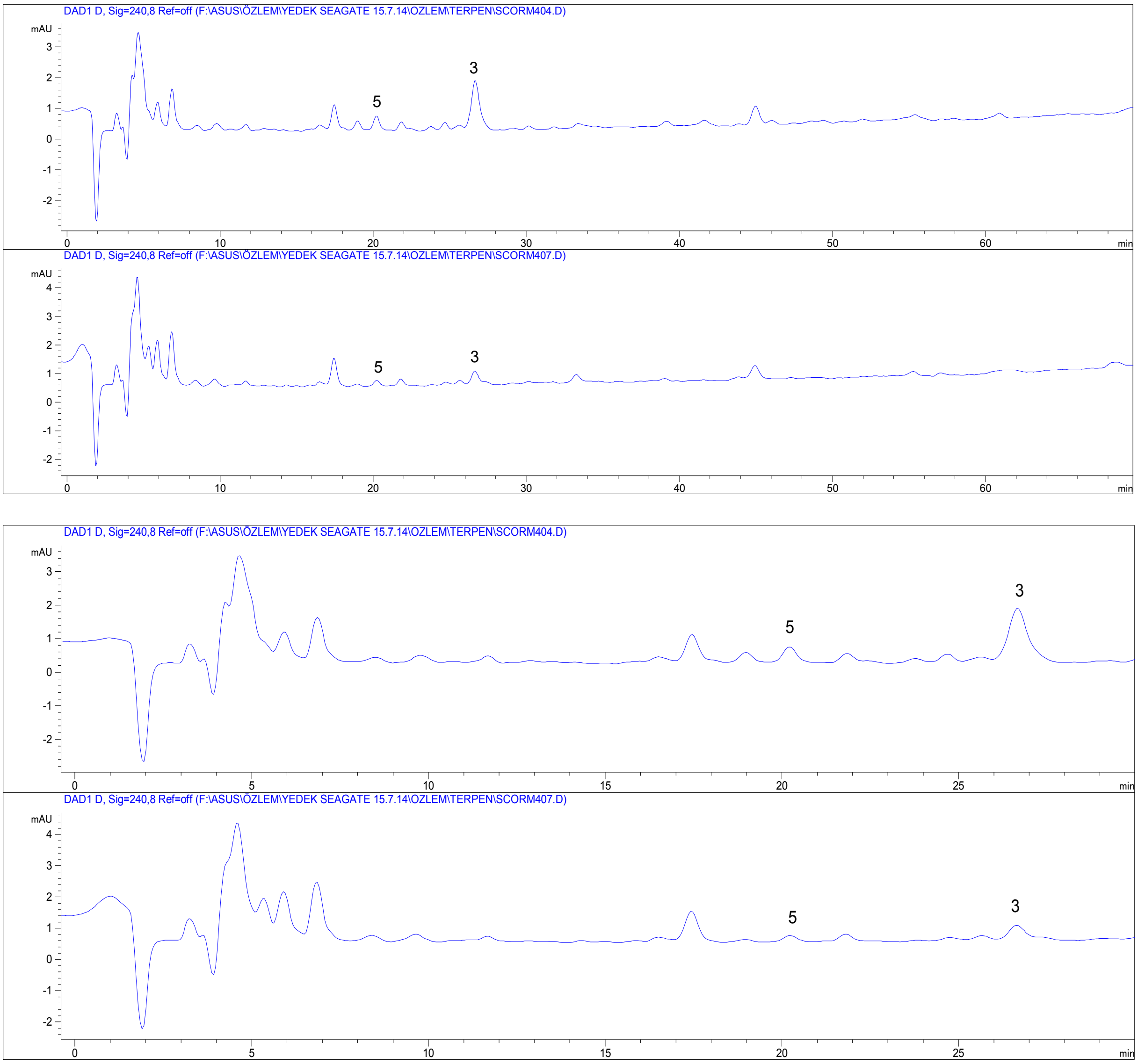

Figure S38: HPLC chromatograms of *S. cana* var. *jacquiniana* root (upper) and aerial (lower) part extract (λ 200 and 240 nm)
